# Use of CRISPR/Cas9 with homology-directed repair to silence the human topoisomerase IIα intron-19 5’ splice site: Generation of etoposide resistance in human leukemia K562 cells

**DOI:** 10.1371/journal.pone.0265794

**Published:** 2022-05-26

**Authors:** Victor A. Hernandez, Jessika Carvajal-Moreno, Xinyi Wang, Maciej Pietrzak, Jack C. Yalowich, Terry S. Elton

**Affiliations:** 1 Division of Pharmaceutics and Pharmacology, College of Pharmacy, The Ohio State University, Columbus, Ohio, United States of America; 2 Department of Biomedical Informatics, College of Medicine, The Ohio State University, Columbus, Ohio, United States of America; Centro de Investigación y de Estudios Avanzados del I.P.N., MEXICO

## Abstract

DNA Topoisomerase IIα (TOP2α/170) is an enzyme essential for proliferating cells. For rapidly multiplying malignancies, this has made TOP2α/170 an important target for etoposide and other clinically active anticancer drugs. Efficacy of these agents is often limited by chemoresistance related to alterations in TOP2α/170 expression levels. Our laboratory recently demonstrated reduced levels of TOP2α/170 and overexpression of a C-terminal truncated 90-kDa isoform, TOP2α/90, due to intronic polyadenylation (IPA; within intron 19) in an acquired etoposide-resistant K562 clonal cell line, K/VP.5. We previously reported that this isoform heterodimerized with TOP2α/170 and was a determinant of acquired resistance to etoposide. Optimization of the weak TOP2α exon 19/intron 19 5′ splice site in drug-resistant K/VP.5 cells by gene-editing restored TOP2α/170 levels, diminished TOP2α/90 expression, and circumvented drug resistance. Conversely, in the present study, silencing of the exon 19/intron 19 5′ splice site in parental K562 cells by CRISPR/Cas9 with homology-directed repair (HDR), and thereby forcing intron 19 retention, was used to induce resistance by disrupting normal RNA processing (i.e., gene knockout), and to further evaluate the role of TOP2α/170 and TOP2α/90 isoforms as resistance determinants. Gene-edited clones were identified by quantitative polymerase chain reaction (qPCR) and verified by Sanger sequencing. TOP2α/170 mRNA/protein expression levels were attenuated in the TOP2α gene-edited clones which resulted in resistance to etoposide as assessed by reduced etoposide-induced DNA damage (γH2AX, Comet assays) and growth inhibition. RNA-seq and qPCR studies suggested that intron 19 retention leads to decreased TOP2α/170 expression by degradation of the TOP2α edited mRNA transcripts. Forced expression of TOP2α/90 in the gene-edited K562 cells further decreased etoposide-induced DNA damage in support of a dominant negative role for this truncated isoform. Together results support the important role of both TOP2α/170 and TOP2α/90 as determinants of sensitivity/resistance to TOP2α-targeting agents.

## Introduction

The human DNA topoisomerase IIα (TOP2α; 170 kDa, TOP2α/170) is an enzyme required to resolve DNA topological entanglements that form during chromosomal condensation, replication, and segregation in proliferating cells [1–4]. TOP2α/170, as a homodimer, creates transient double strand DNA breaks (DSBs) that result in the formation of TOP2α/170-DNA cleavage complexes [1–4]. TOP2α targeting agents (e.g., etoposide, mitoxantrone, m-AMSA, doxorubicin and analogs) stabilize the enzyme-DNA cleavage complexes preventing the religation of DNA; the resultant accumulation of DNA breaks ultimately leads to cell death [2,4–6].

TOP2α poisons are extensively used as chemotherapeutics. However, their efficacy is limited by intrinsic and acquired chemoresistance [7–9]. Acquired resistance to these TOP2α inhibitors is often associated with decreased TOP2α/170 expression levels given that the cytotoxicity of these drugs depends upon the formation and accumulation of TOP2α/170-DNA cleavage complexes [7–9].

Reduction of TOP2α/170 enzyme levels is, in part, associated with intronic polyadenylation (IPA) [10–13] and intron retention [14] which results in the synthesis of C-terminal truncated isoforms of TOP2α. Our laboratory previously demonstrated that acquired resistance to etoposide in a human K562 leukemia cell line, K/VP.5, was associated with decreased TOP2α/170 mRNA/protein expression levels and an increased expression of TOP2α/90 (UniProtKB accession number A0A4D6UXC9), a novel TOP2α isoform that results from intron 19 (I19) IPA [11,15,16]. TOP2α/90 is missing the active site Tyr805 required to form TOP2α–DNA covalent complexes, heterodimerizes with TOP2α/170, and is a determinant of resistance to etoposide [11,15].

IPA is often associated with weak 5′ splice sites (SS), large introns, and strong polyadenylation sites (PAS) (i.e., upstream and downstream *cis* elements including, the AAUAAA hexamer), which regulate RNA cleavage and polyadenylation (polyA) [17,18]. Several studies have established that the strengthening of a weak or suboptimal 5′ SS through mutagenesis could modulate alternative mRNA splicing and IPA [19–25]. Specifically, our laboratory previously improved I19 removal in etoposide resistant K/VP.5 cells [26] by utilizing CRISPR/Cas9 (clustered regularly interspaced short palindromic repeats/CRISPR-associated system 9) with homology-directed repair (HDR) [27–32] to introduce two specific nucleotide changes (GAG//GTAA**AC**→GAG//GTAA**GT**) in the human TOP2α gene’s suboptimal exon 19/intron 19 5′ SS boundary (E19/I19 5′ SS). A gene-edited clone with enhanced I19 removal verified by qPCR and RNA seq, exhibited decreased TOP2α/90 mRNA/protein, and increased TOP2α/170 mRNA/protein expression [26]. This gene edited cell line exhibited increased DNA damage in response to etoposide and other TOP2-targeted drugs as well as increased sensitivity to drug induced-growth inhibition [26]. Together, these results indicated that the optimization of the TOP2α E19/I19 5′ SS in K/VP.5 cells by gene-editing circumvented etoposide resistance and confirmed the importance of RNA processing in acquired drug resistance to TOP2α-targeted drugs [26].

To further establish the role of the E19/I19 5′ SS in drug resistance to TOP2α-targeting drugs, we now report a converse strategy using CRISPR/Cas9/HDR editing of this 5′ SS to repress splicing in parental K562 cells. We hypothesize that silencing the TOP2α E19/I19 5′ SS will result in I19 retention and the subsequent degradation of the TOP2α gene-edited mRNA transcripts (i.e., gene-knockout) since most intron-retaining mRNA transcripts are susceptible to nuclear intron detention/degradation [33] or nonsense-mediated decay (NMD) in the cytoplasm [34]. We further posit that the degradation of the TOP2α E19/I19 5′ SS edited mRNA transcripts will significantly reduce the TOP2α/170 expression levels and subsequently decrease the number of etoposide-induced TOP2α/170-DNA cleavage complexes generated in gene-edited K562 cells; etoposide sensitive cells will then be made drug resistant.

CRISPR/Cas9/HDR editing was utilized to silence the E19/I19 5′ SS by making two changes to the 5′ SS (GA**G**//**G**TAAAC→GA**A**//**C**TAAAC) to prevent spliceosome recognition and induce I19 retention. Two additional mutations (T→G and A→T) were created in the E19/I19 boundary (**T**GA**A**//**C**T**A**AAC→**G**GA**A**/**C**T**T**AAC) to allow for complete discrimination between wild-type cells and CRISPR-edited cells by qPCR (utilizing custom probes) and by restriction digestion analysis. As predicted, silencing the E19/I19 5′ SS resulted in decreased TOP2α/170 mRNA/protein levels, reduced etoposide-induced DNA damage (γH2AX, Comet assays) and generated resistance to etoposide-induced growth inhibition. Forced expression of TOP2α/90 in the gene-edited K562 cells further decreased etoposide-induced DNA damage in support of a dominant negative role for this truncated isoform in the presence of drug. Together, these results demonstrate that CRISPR/Cas9/HDR editing of the TOP2α gene to abrogate normal RNA processing results in etoposide resistance in human leukemia K562 cells and further supports the dual role of TOP2α/170 and TOP2α/90 isoforms as sensitivity/resistance determinants.

## Materials and methods

### Cell culture and drug treatments

Etoposide-resistant K/VP.5 cells were selected and cloned subsequent to intermittent and eventually continuous exposure of K562 cells to 0.5 μM etoposide as previously described [35]. Human leukemia K562, K/VP.5 and gene-edited clonal cells were maintained in DMEM/10% FBS as previously reported [11,15,26]. All experiments described below were performed utilizing cells growing in mid-log phase. Etoposide (2–50 μM) was utilized for short term incubations (30–60 minutes in cell lines. To investigate the potential importance of NMD in the degradation of TOP2α I19 retained mRNA transcripts, K562/SSedit-2 cells were treated with cycloheximide (CHX; i.e., a known indirect inhibitor of NMD [36]) at a final concentration of 100 μM for three hours. Total RNA was isolated as described and qPCR experiments were performed (see below).

### Silencing the TOP2α E19/I19 5′ SS by CRISPR/Cas9/HDR genome editing

Analysis of the wildtype TOP2α E19/I19 5′ SS [37] (TGA**G**//**G**TAAAC; splicing score, 6.1) indicated that, by mutating the two guanine nucleotides (bolded above) in the SS boundary to AC (TGA**A**//**C**TAAAC; splicing score, -7.5), the SS would be eliminated. To simplify and validate the screening of successfully edited clones, two additional mutations (T→G and A→T) were created in the E19/I19 boundary (**T**GA**A**//**C**T**A**AAC→**G**GA**A**//**C**T**T**AAC; splicing score, -11.9) to allow for complete discrimination between wild-type cells and CRISPR-edited cells by qPCR and by generating a MseI restriction enzyme site (T↓TAA). To introduce these four nucleotide changes into the TOP2α E19/I19 5′ SS by HDR, a 180-nucleotide single stranded oligonucleotide (ssODN) repair template (Ultramer Oligo, Integrated DNA Technologies, Coralville, Iowa; see S1 Table for complete sequence) was synthesized and designated “Silenced E19/I19 5′ SS”. This repair template equally spans the TOP2α E19/I19 boundary and harbors the desired four nucleotide changes. Finally, the repair template was chemically modified (phosphorothioate linkages) at the first and last two nucleotides to protect against nuclease degradation.

Genome editing of the TOP2α E19/I19 5′ SS was conducted as follows. Briefly, sgRNA #1 (0.5 μg) was incubated with 2 μg TrueCut Cas9 Protein v2 and 5 μM TOP2α E19/I19 repair template “Silenced E19/I19 5′SS” for 15 min according to the manufacturer’s instructions. This mixture was then transfected into K562 cells (2.25 × 10^6^ cells in 100 μl) by electroporation as reported previously [26]. Forty-eight hours later, K562 cells (1 x 10^6^) were lysed for Cas9 targeting and repair efficiency using the GCD assay described above. After verification of successful on-target genome editing generated by non-homologous end joining (NHEJ), the remaining transfected K562 cells were plated using limiting dilution cloning in five 96 well plates (0.8 cells per well). Aliquots (~25–50,000 cells) from single cell clones were subsequently lysed with GCD buffer (see below) ~2 weeks after plating. Supernatants were assayed for HDR by genomic qPCR using a validated wild-type E19/I19 qPCR hybridization probe (5’-TCATGGTGAG//GTAAACACACAATCC-3’) [11,15,26] and a mut/E19/I19 hybridization probe specific for the edited TOP2α E19/I19 5’ SS (5’-TCATGGGGAA//CTTAACACACAATCC-3’) to identify colonies with at least one TOP2α edited allele. Note that there are three TOP2α alleles present in the K562 cell line [38,39]. After transfection, multiple colonies with one and two edited allele(s) were identified by qPCR (see procedure below) and confirmed by sequencing and MseI restriction enzyme analysis (see below).

### Genomic Cleavage Detection (GCD)

A custom single-piece TrueGuide RNA, TOP2α sgRNA-1 (5’-GTCTTCTTATCATCATGGTG-3’), was obtained from ThermoFisher. The TrueGuide RNA is chemically modified (2´O-Methyl analogs and phosphorothioate linkages) to increase editing efficiency and protect against nuclease degradation. The sgRNA-1 (0.5 μg) was incubated with 2 μg TrueCut Cas9 Protein v2 (cat. no. A36498; ThermoFisher) for 15 minutes to form Cas9 protein/gRNA ribonucleoprotein complexes. These complexes were subsequently transfected into K562 cells by electroporation technology (Nucleofector Kit V; Lonza, Basel, Switzerland) according to manufacturer’s instructions and as reported previously [11,15,26].

To determine if the gene-specific Cas9 protein/gRNA ribonucleoprotein complexes created on-target DSBs within the TOP2α E19/I19 boundary sequence, K562 cells (2 x 10^6^) were lysed 48 hours after transfection using cell lysis GCD buffer/Proteinase K (GeneArt Genomic Cleavage Detection Kit (cat. no. A24372; ThermoFisher). Genomic DNA (1 μL of lysate) at the TOP2α locus from exon 18 (E18) through I19 was then PCR amplified (50 μL reaction volume) using the following primers: GCD TOP2α E18 For (5’-GATCTATCCCTTCTATGGTGG-3’) and GCD TOP2α I19 Rev (5’-CAGAAATCAAAGGGCAAGCAG-3’). The PCR amplicons were subsequently denatured, reannealed and incubated with T7 endonuclease I (i.e., structure-selective enzyme that recognizes and cleaves mismatched DNA) to detect insertions/deletions (INDELs) created by NHEJ. The digested and non-digested PCR products were fractionated by electrophoresis on a 2% agarose gel and images were captured as described above. The following equation was used to the calculate the cleavage efficiency of TOP2α gRNA-1/Cas9: Cleavage Efficiency = {1– [(1–Fraction Cleaved)^½^]} x 100, where fraction cleaved = (sum of cleaved band intensities)/(sum of the cleaved and parental band intensities) [32].

### MseI restriction enzyme analysis of CRISPR/Cas9 edited K562 cells

K562 cells and the selected clones denoted K562/SSedit-1, and K562/SSedit-2 were lysed using cell GCD lysis buffer/Proteinase as described above. Genomic DNA (1 μl of lysate) at the TOP2α locus between E18/I18/E19/I19 was subsequently PCR amplified (50 μl rxn volume) using the following primers: GCD TOP2α E18 For (5’-GATCTATCCCTTCTATGGTGG-3’) and GCD TOP2α I19 Rev (5’-CAGAAATCAAAGGGCAAGCAG-3’). Ten μL of the PCR reaction was then digested by MseI (cat. no. R0525S, New England Biolabs) according to manufacturer’s instructions. The digested and non-digested PCR products were fractionated by electrophoresis on a 2% agarose gel and images were captured as described above.

### Quantitative real-time PCR assays

Total RNA was isolated from K562, K/VP.5, and CRISPR/Cas9 edited K562/SSedit-1 and K562/SSedit-2 cells using the RNA Easy Plus Mini Kit (cat. no.74134; Qiagen, Germantown, MD). To ensure complete removal of contaminating DNA, an on-column digestion of DNA with RNase-free DNase (cat. no.79254; Qiagen) was included during RNA purification. RNA (1 μg) was reverse transcribed using random hexamers and MultiScribe Reverse Transcriptase (High-Capacity cDNA Reverse Transcription Kit, cat. no. 4368814; ThermoFisher Scientific, Waltham, MA) as previously described by our laboratory [11,15,26]. Quantitative real-time PCR experiments (total reaction volume 10 μL) were performed using TaqMan Gene Expression hydrolysis probes (ThermoFisher Scientific) [11,15,26]. TOP2α/170 mRNA expression levels were measured using a hydrolysis probe spanning the TOP2α E19/E20 boundary (5’-TCATGGTGAG//ATGTCACTAATGATG-3’) (TaqMan assay Hs01032135_m1) specific for TOP2α/170 cDNAs. TOP2α I19 retained mRNA expression levels were measured using hydrolysis probes spanning the TOP2α E19/I19 boundary using either a qPCR hybridization probe containing the gene-edited sequences (5’-TCATGGGGAA//CTTAACACACAATCC-3’) or a hydrolysis probe spanning the TOP2α I19/E20 boundary (5ʹ-TTTTTTTCCCCACAG//ATGTCACTAATGATG-3ʹ). SRSF3 transcript variant-1 (NM_003017.5, not subject to NMD [40,41]) mRNA expression levels were measured using TaqMan assay Hs01120547_g1. SRSF3 transcript variant-2 (NR_036610.2, subject to NMD [40,41]) mRNA expression levels were measured using TaqMan assay Hs01122146_g1. The expression levels mRNAs for TOP2α/170, wildtype TOP2α E19/I19, gene-edited TOP2α mut E19/I19, TOP2α I19/E20, SRSF3 (variant-1), and SRSF3 (variant-2) were normalized to TATA-box binding protein (TBP, TaqMan assay Hs99999910_m1) expression using the 2^−ΔΔCt^ method [42].

### Immunoassays

Extracts from K562, K/VP.5, and CRISPR/Cas9 edited K562/SSedit-1, and K562/SSedit-2 cells (± etoposide treatment) were subjected to Western blot analysis as previously described [11,15,26]. Unless otherwise noted, 16 *μ*g of protein was loaded into each well. Membranes were incubated overnight at 4°C with one of the following primary antibodies: a rabbit polyclonal antibody raised against the human TOP2*α*/170/90 N-terminal sequence (amino acids 14–27) (cat. no. ab74715; Abcam, Cambridge, MA; used at 1:1000 dilution), an anti-rabbit TOP2α/170 C-terminal specific antibody (generated against a recombinant 70-kDa fragment corresponding to only the TOP2α/170 C-terminal sequence [35]; used at 1:5000 dilution), a mouse monoclonal glyceraldehyde 3-phosphate dehydrogenase (GAPDH) antibody (cat. no. sc-47724; Santa Cruz Biotechnology, Santa Cruz, CA; used at 1:5000 dilution), a mouse γH2AX (phosphorylated Ser-139 residue of the H2A histone family member X) monoclonal antibody (cat. no. sc-25330; Santa Cruz Biotechnology; used at 1:500 dilution). The membranes were subsequently incubated at room temperature for ∼3 hours with a donkey anti-rabbit or anti-mouse secondary antibody (1:5000 dilution) (Jackson Immuno Research, West Grove, PA). Finally, TOP2*α* isoforms, GAPDH, and γH2AX were detected using the Clarity Max chemiluminescence kit (Bio-Rad Laboratories, Hercules, CA). All immunoassay images were acquired with the ChemiDoc XRS+ imaging system and analyzed with ImageLab software (Bio-Rad Laboratories).

### Growth inhibitory assays

Growth inhibitory assays were performed as previously described [11,15,26]. Briefly, log-phase K562 cells, K/VP.5 cells, and gene-edited K562 clonal cells were adjusted to 1–1.5 X 10^5^ cell/mL and incubated for 48 hours with DMSO as control solvent (final concentration 0.5%) and with various concentrations of etoposide, after which cells were counted on a model Z1 DUAL Coulter counter (Beckman Coulter, Indianapolis, IN). The extent of growth beyond the starting concentration in drug-treated versus control cells was expressed ultimately as percent inhibition of control growth. The 50% growth inhibitory values for etoposide and each cell line were derived from replicate experiments performed on separate days fitting the concentration-response (inhibition) curves to a four-parameter logistic equation using Sigmaplot 14.5 (Systat Software, Inc., San Jose, CA).

### DNA damage (Comet) assays

Alkaline (pH 13, detects primarily single-strand breaks, SSBs) single-cell gel electrophoresis (Comet) assays were performed according to the manufacturer’s protocol (CometAssay Kit, cat. no. 4250–050 K; Trevigen, Gaithersburg, MD) and as previously described by our laboratory [11,15,26]. Briefly, K562, K/VP.5, K562/SSedit-1, and K562/SSedit-2 cells were washed and resuspended in buffer (25 mM HEPES, 10 mM glucose, 1 mM MgCl_2_, 5 mM KCl, 130 mM NaCl, 5 mM NaH_2_PO_4_, pH 7.4). Cells were subsequently incubated with 2 or 10 μM etoposide or DMSO (solvent control) for 30 minutes at 37°C. The treated cells were washed with ice-cold buffer and resuspended to 0.28 × 10^6^ cells/ml and then further diluted in low melt agarose. Following alkaline electrophoresis (of ~2000 cells) and subsequent staining with a fluorescent DNA intercalating dye, SYBR Gold, the migrating fragments (comet tail) from the nucleoid (comet head) were visualized and the images captured by fluorescence microscopy. The Olive tail moment [43], was quantified by the ImageJ processing program with the open-source software tool OpenComet [44; www.cometbio.org]. Olive tail moments from greater than 100 cells per sample condition were determined.

### Forced expression of TOP2α/90

K562 cells (2.25 x 10^6^ cells in 1 ml per condition) were mock transfected (negative control) or transfected with the pcDNA3.4/TOP2α/90 construct [11,15] (10 μg plasmid) by electroporation technology (Nucleofector Kit V; Lonza, Basel, Switzerland), according to manufacturer’s instructions. Forty-eight hours after transfection, cellular extracts were prepared and immunoblotting experiments were performed, as outlined above, utilizing the TOP2*α*/170/90 N-terminal specific antibody. In addition, pcDNA3.4/TOP2α/90 transfected K562 cells were utilized for comet assays as described above.

### RNA sequencing and bioinformatics analyses

DNA-free RNA was isolated from K562, K/VP.5, and K562/SSedit-2 cells using the RNA Easy Plus Mini Kit (cat. no. 74134; Qiagen, Germantown, MD) with on-column digestion with RNase-Free DNase (cat. no. 79254). Two rounds of purification were used to assure the integrity of the isolated total RNA. RNA sequencing (RNA-seq) libraries were prepared at The Ohio State University Comprehensive Cancer Center Genomics Shared Resource and sequenced from quadruplicate samples from each cell line. Paired-end RNA-seq was performed on an Illumina HiSEq 4000 platform at the Genomics Services Laboratory of The Research Institute at Nationwide Children’s Hospital, Columbus, OH. Illumina 150-bp paired-end RNA-seq raw reads from K562, K562/SSedit-2, and K/VP.5 RNA samples were mapped to the human reference genome GRCh38 using Hierarchical Indexing for Spliced Alignment for Transcripts version 2.1.0 9 [45], converted to bigwig coverage tracks using deepTools [46], and visualized using the Integrative Genomics Viewer [47]. Gene counts were generated with featureCounts [48] as described in Gadepalli et al. [49]. Gene expression was quantified as log2 counts per million, and differential expression analysis was performed using R limma voom function [50]. RNA-seq data are available in the Gene Expression Omnibus with accession number (GSE200926).

### Data analysis

Statistical analysis was performed using SigmaPlot 14.5. All data were expressed as the mean ± standard deviation (SD). Unless noted otherwise, group-wise differences were analyzed using a two-tailed paired Student’s t test with no adjustment for multiple comparisons. A P-value of <0.05 was considered statistically significant with 95% confidence intervals (CI) noted in Figure legends.

## Results

### CRISPR/Cas9/HDR: Strategy to silence the TOP2α E19/I19 5′ SS in K562 cells

**Fig 1:**
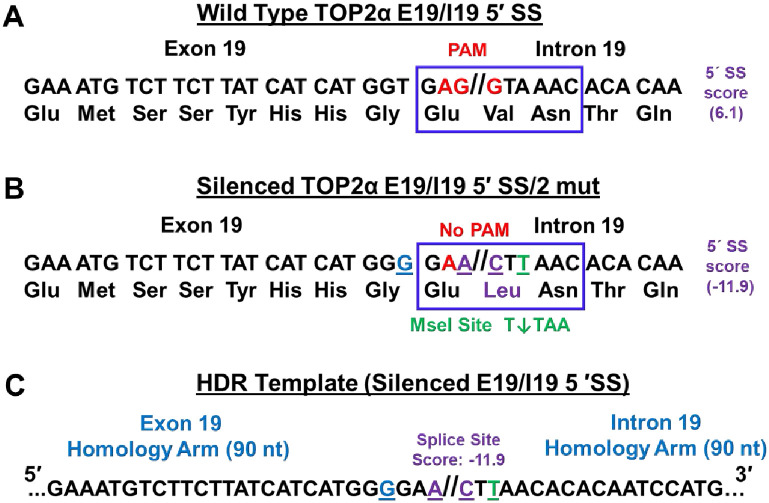
Strategy to utilize CRISPR/Cas9/HDR to silence the TOP2α E19/I19 5′ SS in K562 cells. **(A)** The TOP2α E19/I19 gene boundary sequence is shown. The wild-type E19/I19 5′ SS sequence to be edited by CRISPR/Cas9 is boxed in blue. The SS splicing score is also denoted. The PAM site is denoted in red. **(B)** The proposed two sequence changes to “silence” the E19/I19 5′ SS (boxed in blue) are underlined and denoted in purple (G//G→A//C). The G→A edit is synonymous and would not result in an amino acid change in the TOP2α/170 protein. The G→C edit is nonsynonymous and would result in an amino acid change (Val→Leu, shown in purple) in the TOP2α/90 isoform if expressed. The “silenced” 5′ SS splicing score is also denoted. The proposed sequence changes to “silence” the E19/I19 5′ SS also eliminates the PAM site (AGG→AAC). Additional editing of the last nucleotide of the triplet codon for Leu (CTA→CTT denoted in green and underlined) introduces a restriction site for the MseI endonuclease (T↓TAA). Successful TOP2α gene editing would allow for MseI digestion at the E19/I19 boundary. The triplet GGT codon upstream from 5′ SS was also edited to a GGG, a synonymous change denoted in blue, to allow for greater discrimination between the wild-type and edited E19/I19 boundary using a specific qPCR probe. **(C)** To introduce the proposed changes (underlined and denoted in color) in the TOP2α E19/I19 5′ SS, a ssODN HDR template (denoted “silenced E19/I19 5′ SS”) was synthesized.

The wild-type TOP2α E19/I19 5′ SS (GAG//GTAAAC) (Fig 1A) was analyzed to determine the impact of specific gene edits on the 5’ SS scores [37]. This analysis indicated that mutating the two guanine nucleotides (G//G→A//C, shown in purple and underlined; Fig 1B) in K562 cell TOP2α, that denote the boundary between E19 and I19, would decrease the splicing score of 6.1 (wild-type TOP2α E19/I19 5′ SS) to a splicing score of -7.5 (not shown) [37] and likely eliminate the ability of the splicing machinery to remove TOP2α I19. As a consequence of hypothesized I19 retention and subsequent degradation [33,34] of the TOP2α E19/I19 5′ SS edited mRNA transcripts, TOP2*α*/170 would be expected to be decreased (i.e., gene-knockout). Hence, it was anticipated that CRISPR/Cas9/HDR TOP2α-edited parental K562 cells would become etoposide resistant due to the decrease in TOP2*α*/170 expression and the resulting reduction in etoposide stabilized TOP2α/170-DNA cleavage complexes [1–4].

Within the E19/I19 5′ SS, the first guanine nucleotide substitution (G→A) would result in a synonymous mutation and the TOP2α/170 protein sequence would be unchanged (Fig 1B). The second guanine nucleotide substitution (G→C) would result in a nonsynonymous mutation altering one amino acid sequence (Val→Leu denoted in purple) in only the TOP2α/90 isoform (Fig 1B). Additionally, sequence changes to “silence” the E19/I19 5′ SS would also eliminate a critical protospacer-adjacent motif (PAM) site (AGG→AAC) given that it is located exactly at the boundary between E19 and I19 (Fig 1B). It was expected that clonal cell lines with one or two TOP2α alleles edited would be identifiable. It was also anticipated that clones with all three TOP2α alleles (K562 cells are trisomic [38,39]) silenced would not (and could not) be isolated since the lack of TOP2α/170 expression would be lethal given that this enzyme is essential for cell viability [51].

To facilitate and validate the screening of successfully edited clones, two additional silent mutations (T→G [denoted in blue] and A→T [denoted in green]) were generated in the E19/I19 boundary to allow for complete discrimination between wild-type K562 cells and CRISPR/Cas9/HDR edited K562 cells by qPCR, and to create a MseI restriction enzyme site (T↓TAA), resulting in a further reduction in the splicing score to -11.9 (Fig 1B). A symmetric 180 nucleotide ssODN HDR template (denoted as “Silenced E19/I19 5′ SS”) with 90 nucleotide homologies to both TOP2α E19 and I19 and harboring the four 5’ SS nucleotide changes (underlined and denoted in color; Fig 1C) described above, was synthesized and utilized in all CRISPR/Cas9/HDR transfection experiments.

### sgRNA-1 directs Cas9 cleavage in the TOP2α E19/I19 boundary sequence

**Fig 2:**
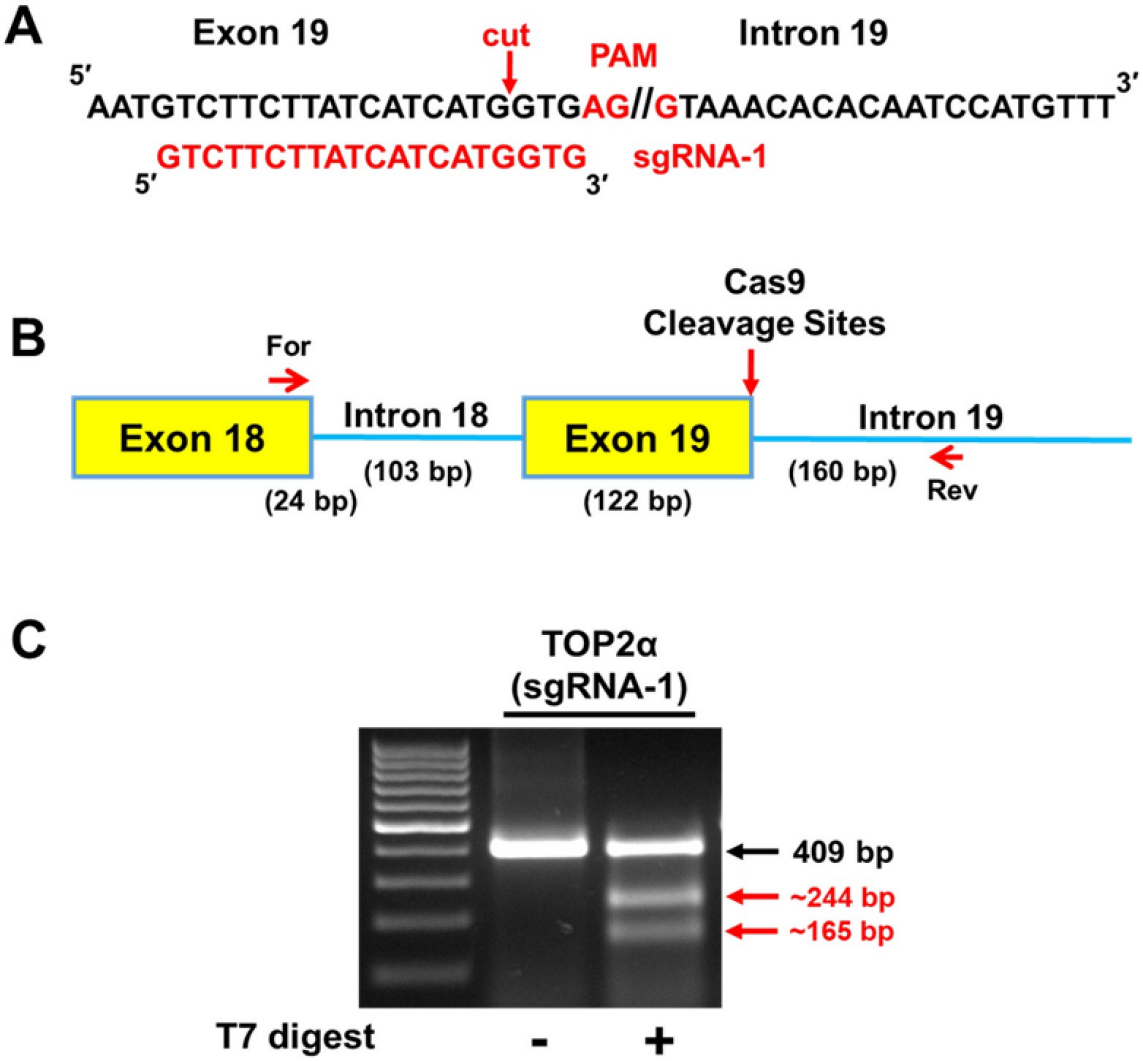
Assessment of sgRNA-1/Cas9 complex cleavage efficiency at the TOP2α E19/I19 boundary sequence. **(A)** Sequence of the TOP2α E19/I19 boundary is shown. PAM/sgRNA-1 site is shown in red. The red arrow denotes where Cas9 will generate a DSB. **(B)** Schematic representation of the E18 through I19 region of the TOP2α gene. The larger red arrow denotes the site where sgRNA-1 directed Cas9 cleavage and NHEJ generation of INDELs can occur. The small red arrows denote the forward and reverse primers used for the GCD assay. **(C)** Ethidium bromide-stained agarose gel fractionated GCD PCR amplicons before and after treatment with T7 endonuclease I. The parental and T7 endonuclease I cleaved daughter PCR amplicons are indicated and their respective sizes are denoted.

The CRISPR/Cas9 system [27–32] was utilized to introduce specific gene edits in the TOP2α E19/I19 5′ SS in K562 cells through HDR [26] to silence the 5′ SS and prevent the splicing out of I19 (i.e., increase I19 retention and subsequent degradation of mRNA transcripts transcribed from the edited TOP2α alleles). The gRNA/Cas9 complex binds to the target site and the Cas9 nuclease introduces a blunt-end DSB three bases upstream of the PAM site [27,28]. The TOP2α E19/I19 boundary sequence (200 base pairs) was previously analyzed for PAM sequence motifs utilizing the CRISPR/Cas9 target online predictor (CCTop) [52]. Three candidate PAMs in close proximity to the intended mutations were previously examined using two-piece guide RNAs [26]. For the present study, a single-piece gRNA-1 (sgRNA-1) (Fig 2A) was utilized to eliminate the required annealing step of the trans-activating CRISPR RNA (tracrRNA) and crispr RNA (crRNA) to form a tracrRNA/crRNA hybrid (i.e., two-piece guide RNAs) and thus increase the efficiency of the sgRNA to target the Cas9 to the appropriate cleavage site [31].

To determine the Cas9 cleavage efficiency at the PAM/sgRNA-1 site (Fig 2A), sgRNA-1 was combined with Cas9 and transfected into K562 cells. Forty-eight hours post transfection, cells were assessed for INDELs due to NHEJ [53] and Cas9 cleavage in the sequence covering TOP2α E19/I19. Briefly, GCD buffer, followed by PCR using an E18 forward primer and an I19 reverse primer to amplify the CRISPR/Cas9 target region (Fig 2B). Amplicons were denatured and re-annealed to evaluate Cas9-induced breaks. Mismatches were subsequently cleaved by T7 endonuclease I. Results revealed that PAM/sgRNA-1 effectively guided Cas9 to the target site (Fig 2C). The calculated Cas9 cleavage efficiency [32] in the TOP2α E19/I19 boundary by the TOP2α sgRNA-1 was 21.4%. The efficiency of sgRNA-1 was much higher than the efficiency of a 2-piece gRNA-2 (7.3%) utilized previously [26].

### CRISPR/Cas9/HDR: qPCR selection and sequence analysis of Edited TOP2α E19/I19 5′ SS clonal cell lines

**Fig 3:**
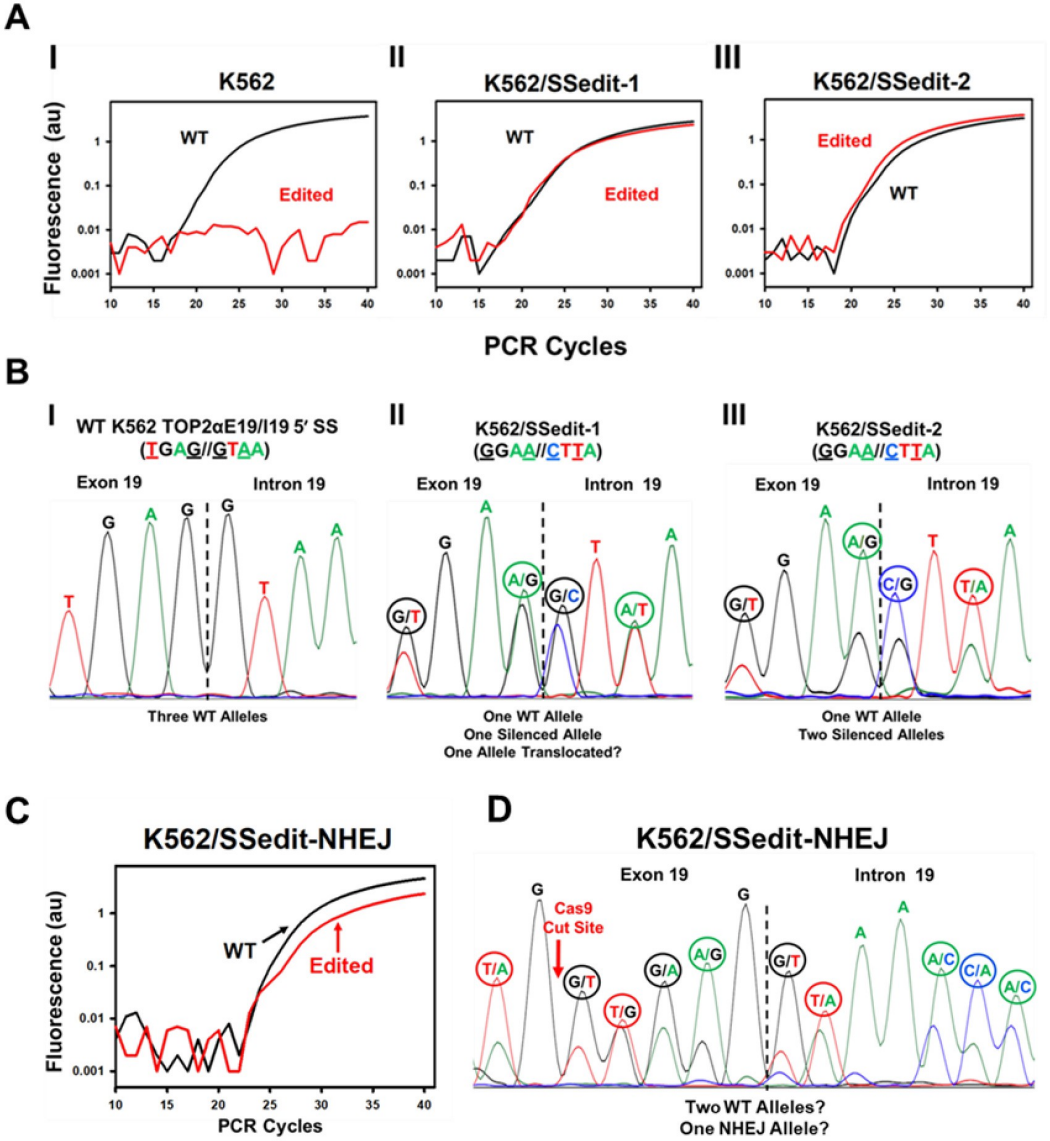
Selection of CRISPR/Cas9/HDR edited TOP2α E19/I19 5′ SS clonal cell lines. **(A)** Amplification plots of qPCR reactions from K562, K562/SSedit-1, and K562/SSedit-2 cells (labeled I-III) using wild-type TOP2α E19/I19 and edited-specific mut/E19/I19 boundary qPCR probes. **(B)** Electropherogram of the genomic sequence of the TOP2α E19/I19 gene boundary in K562, K562/SSedit-1, and K562/SSedit-2 cells (labeled I-III). **(C)** Amplification plots of genomic qPCR reactions from K562/SSedit-NHEJ cells using E19/I19 and mut/E19/I19 boundary qPCR probes. **(D)** Electropherogram of the genomic sequence of the TOP2α E19/I19 gene boundary in K562/SSedit-NHEJ cells.

K562 cells were transiently transfected (48 hours) with sgRNA-1 (Fig 2A and S1 Table), Cas9 protein, and the HDR template (Fig 1C). Cells were then seeded at 0.8 cell/well in 96 well plates. Two weeks later, cells harvested from single-colony wells were lysed and screened for E19/I19 editing by genomic DNA qPCR [26]. To discriminate between the wild-type TOP2α E19/I19 and the CRISPR/Cas9/HDR edited TOP2α E19/I19 boundary, a previously validated wild-type E19/I19 qPCR hybridization probe (5’-TCATGGTGAG//GTAAACACACAATCC-3’) [11,15,26] and a unique mut/E19/I19 qPCR hybridization probe containing the nucleotides to be edited (5’-TCATGGGGAA//CTTAACACACAATCC-3’) were used. In non-transfected K562 cells, a qPCR signal was not observed when the mut/E19/I19 probe was utilized (Fig 3A-I, red line). However, the wild-type TOP2α E19/I19 boundary probe (black line) yielded a positive genomic qPCR signal (Fig 3A-I). Sanger sequencing verified the wild-type TOP2α genomic sequence (Fig 3B-I).

In contrast, when CRISPR/Cas9/HDR transfected K562 cell lysates were screened (~25 clonal cell colonies), several clones were identified where the qPCR tracing for the TOP2α mut/E19/I19 boundary probe resulted in a positive signal (red line) as exemplified by a clone denoted K562/SSedit-1 (Fig 3A-II). This clone exhibited a qPCR mut/E19/I19 boundary probe signal (red line) that overlayed almost precisely with that of the wild-type E19/I19 boundary probe tracing (black line, Fig 3A-II). Sanger sequence analysis of K562/SSedit-1 demonstrated that both the wild-type and edited genomic sequences were present in the TOP2α E19/I19 boundary at all four of the edited sites (Fig 3B-II). Although K562 cells contain three TOP2α alleles [38,39], the Sanger sequencing electropherogram signals for wild-type and edited bases were nearly one-to-one (Fig 3B-II). These results suggested that only one of the three TOP2α E19/I19 alleles contained the desired gene edits in this clonal cell line, while one allele remained unchanged, and very likely the third allele underwent a translocation. Therefore, further characterization of K562/SSedit-1 cells was limited.

Additional qPCR screening led to the identification of a CRISPR-edited clone, designated K562/SSedit-2. The genomic DNA qPCR amplification plots of K562/SSedit-2 cells indicated that signal from the mut/E19/I19 qPCR probe appeared ~one PCR cycle earlier than the signal from the wild-type E19/I19 qPCR probe (Fig 3A-III), suggesting that two TOP2α alleles were edited and one TOP2α allele was unchanged. Sanger sequence analysis of K562/SSedit-2 corroborated the qPCR results, demonstrating that two alleles contained all four of the mutated sites of the TOP2α E19/I19 boundary in a ratio of two-to-one compared to the wild-type TOP2α sequence (Fig 3B-III).

Of note, most of the single cell colonies screened after transfection of K562 cells with Cas9/sgRNA-1 and repair template yielded qPCR amplification plots that suggested one TOP2α allele had been edited. An example is shown in Fig 3C. However, genomic sequencing revealed that these clones had undergone NHEJ at one allele since multiple unintended nucleotide changes were observed in the sequencing electropherogram signals (i.e., the smaller peaks), most of which appeared to be present at a ratio of one-to-two compared to the wild-type TOP2α E19/I19 boundary signal (Fig 3D). These clones were not characterized further.

### MseI analysis validation of CRISPR/Cas9/HDR editing of K562/SSedit-2 cells

**Fig 4:**
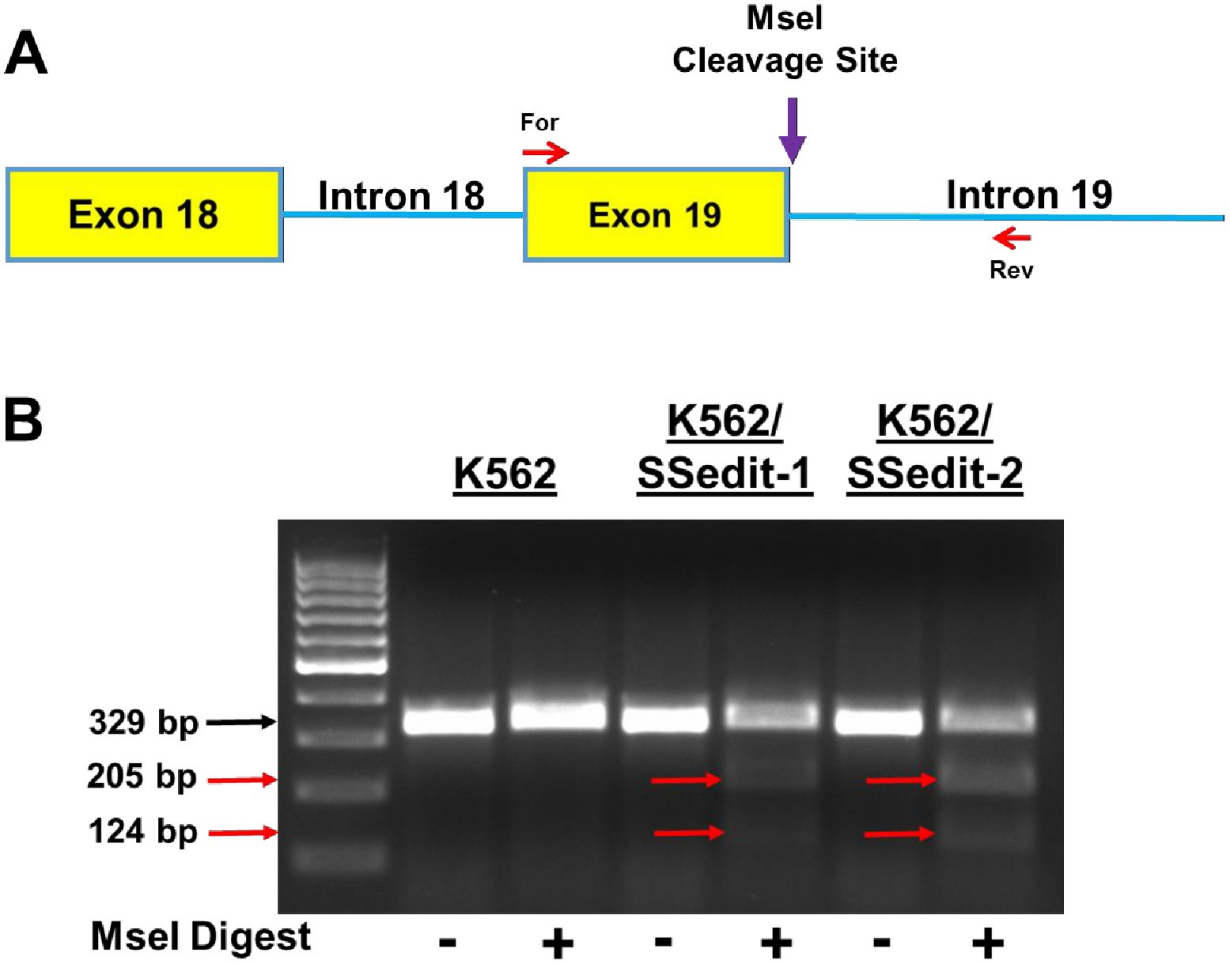
MseI validation of CRISPR/Cas9 edited TOP2α E19/I19 5′ SS clonal cell lines. **(A)** Schematic representation of the E18 through I19 portion of the TOP2α gene. Red arrows denote the forward and reverse primers used for the identification of CRISPR editing of the TOP2α E19/I19 5′ SS using MseI endonuclease **(T↓TAA)**. The purple arrow denotes site where MseI creates double strand breaks of the CRISPR-edited PCR amplicon. **(B)** Ethidium bromide-stained agarose gel of fractionated MseI-treated PCR amplicons from K562, K562/SSedit-1 and K562/SSedit-2 cells DNA. The black arrow denotes the parental PCR amplicon. Expected sizes of daughter PCR amplicons are indicated by red arrows.

Gene editing the 3rd nucleotide within the I19 sequence (A→T) (Fig 1A and 1B) introduced a restriction site for the MseI endonuclease (T↓**T**AA; Fig 1B). Successful TOP2α gene editing would allow for MseI digestion at the E19/I19 boundary. Thus, an independent assay with MseI endonuclease was carried out to validate correct CRISPR-editing of the TOP2α gene. Cell lysates from K562 cells and the CRISPR-generated cell lines (K562/SSedit-1 and K562/SSedit-2) were used as templates for PCR reactions; forward primer annealing to TOP2α E18; reverse primer annealing to TOP2α I19 (Fig 4A). Based on the location of the forward and reverse primers, the expected size of the parental PCR amplicon was 329 bp. After MseI digestion, the expected sizes of putative gene edited clones were 205 bp and 124 bp (Fig 4B). As shown, MseI did not result in cleavage in non-edited K562 cells (Fig 4B). In contrast, partial MseI digestion of the 329 bp band was evident in K562/SSedit-1 and K562/SSedit-2 lysates with reduction in the parental amplicon. After incubation with MseI, the percent cleavage in K562/SSedit-1 and K562/SSedit-2 PCR amplicons was 8.3% and 17.3%, respectively. These results further confirmed that K562/SSedit-1 cells have one successfully edited TOP2α allele, while K562/SSedit-2 cells have two edited TOP2α alleles.

### TOP2α/170 and TOP2α/90 Expression in K562/SSedit-1 and K562/SSedit-2 cells

**Fig 5:**
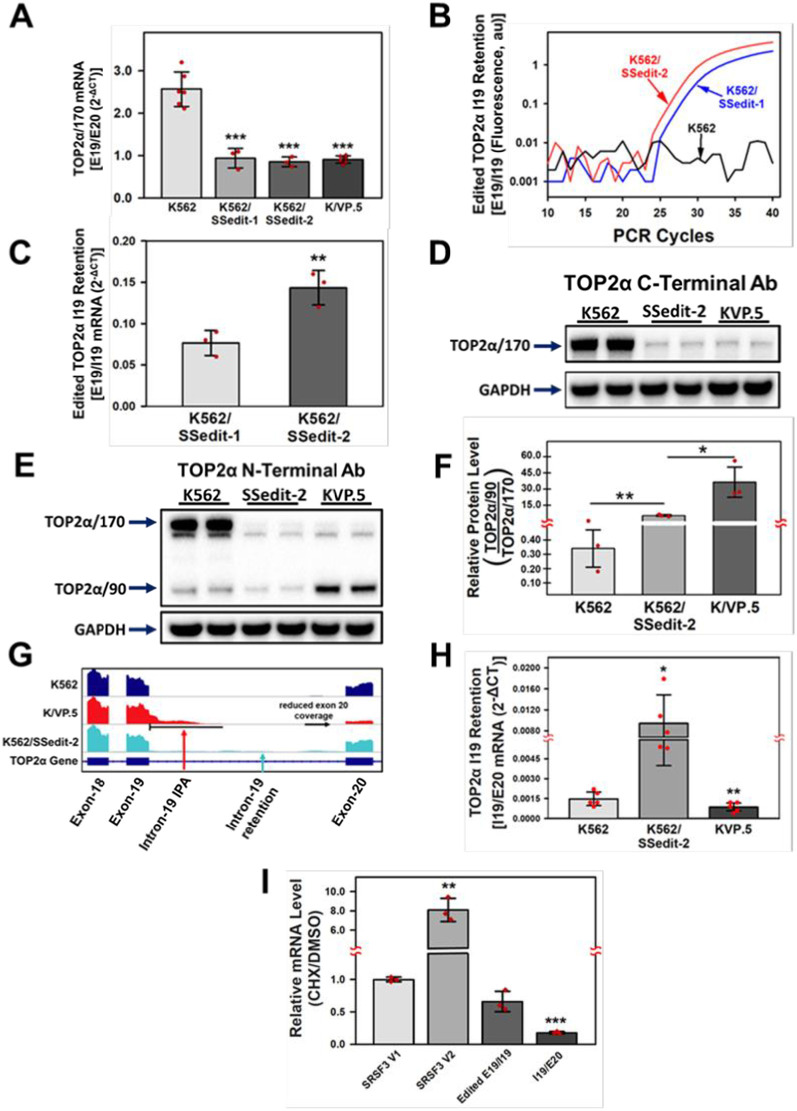
TOP2α/170 mRNA and protein levels in K562, drug resistant K/VP.5, and K562/SSedit-2 cells. **(A)** qPCR experiments were performed utilizing K562, K/VP.5, K562/SSedit-1 and K562/SSedit-2 cDNAs and a TaqMan hydrolysis assay specific for TOP2α/170 (E19/E20) mRNAs. Results shown are the mean ± SD from three to six experiments run on separate days in which RNA/cDNA isolations/determinations were performed. Using a two-tailed Student’s *t* test comparing the differences in mean calculated 2^-ΔCt^ values: *P* <0.001 for K/VP.5 vs. K562 TOP2α/170 mRNA, 95% CI (-2.04, -1.28); *P* < 0.001 for K562/SSedit-1 vs. K562 TOP2α/170 mRNA, 95% CI (-2.24, -1.02); *P* < 0.001 for K562/SSedit-2 vs. K562 TOP2α/170 mRNA, 95% CI (-2.30, -1.13). **(B)** Amplification plots of qPCR reactions of cDNAs from K562, K562/SSedit-1, and K562/SSedi-2 cells using edited-specific mut/E19/I19 boundary qPCR probes. **(C)** qPCR of edited TOP2α I19 retained cDNAs from K562/SSedit-1 and K562/SSedit-2 cells probed with a TaqMan hydrolysis assay specific for the mut/E19/I19 boundary containing the four base-pair changes. Results shown are the mean ± SD from three paired RNA/cDNA isolations/determinations performed on separate days; *P* <0.01, comparing the differences in mean calculated 2^-ΔCt^ values for K562/SSedit-2 vs. K562/SSedit-1 edited TOP2α I19 retained mRNA, 95% CI (0.052, 0.081). **(D)** Representative immunoassay using K562, K/VP.5, and K562/SSedit-2 cellular lysates. Blots were probed with antibodies specific for the C-terminal portion of TOP2α/170 (C-terminal amino acids) or for GAPDH. **(E)** Representative immunoassay using K562, K/VP.5, and K562/SSedit-2 cellular lysates. Blots were probed with antibodies specific for the N-terminal portion of TOP2α/170/90 (i.e., amino acids 14–27) or for GAPDH. **(F)** The ratio of TOP2α/90-to-TOP2α/170 protein expression levels from K562, K/VP.5, and K562/SSedit-2 cellular lysates. Results shown are means ± S.D. from three experiments performed on separate days; P < 0.01 comparing the difference in mean values for K562/SSedit-2 and K562 cells TOP2α/90 ratios to TOP2α/170, 95% CI (3.02, 7.25). P < 0.05 comparing the difference in mean values for K/VP.5 and K562/SSedit-2 cells TOP2α/90 ratios to TOP2α/170, 95% CI (-13.67, 75.57). **(G)** Visualization of TOP2α RNA-seq genome coverage tracks from exon 18 through exon 20 in K562, K/VP.5, and K562/SSedit-2 cells. K/VP.5 cell I19 IPA (underlined in black/denoted with a red arrow) results in increased TOP2α/90 protein expression; see Fig 5E). Subsequent reduced coverage for E20 in K/VP.5 cells (underlined in black/denoted with a red arrow) is consistent with decreased TOP2α/170 protein expression levels (see Fig 5E). K562/SSedit-2 cell I19 retention is labeled and denoted with a light blue arrow. I19 IPA is not observed in K562/SSedit-2 cells, consistent with low TOP2α/90 protein expression observed in these cells (Fig 5E). I19 IPA and I19 retention is not observed in parental K562 cells. **(H)** qPCR experiments were performed utilizing K562, K/VP.5 and K562/SSedit-2 cDNAs and a TaqMan hydrolysis assay specific for I19 retaining mRNAs (I19/E20 boundary). Results shown are the mean ± SD from five paired RNA/cDNA isolations/determinations performed on separate days; P = 0.02 comparing the differences in mean calculated 2^-ΔCt^ values for K562/SSedit-2 vs. K562 TOP2α I19 retention, 95% CI (0.002, 0.014). P = 0.006, comparing the differences in mean calculated 2^-ΔCt^ values for K/VP.5 vs. K562 TOP2α I19 retention, 95% CI (-0.0009, -0.0003); **(I)** qPCR experiments were performed utilizing K562/SSedit-2 cDNAs synthesized from cells treated with DMSO solvent or 100 μM CHX for 3 hours using the TaqMan hydrolysis assays as denoted in Methods. Results shown are the mean ± SD from three paired RNA/cDNA isolations/determinations performed on separate days normalized to the NMD negative control (SRSF3 V1) ratio of CHX to DMSO treatment; P = 0.009, comparing the differences in mean CHX/DMSO ratios for NMD positive control (SRSF3 V2) vs. NMD negative control (SRSF3 V1), 95% CI (2.76, 6.49); P < 0.001, comparing the differences in mean CHX/DMSO ratios for I19/E20 mRNA transcripts vs. NMD negative control (SRSF3 V1) 95% CI (-0.86, -0.78). *P < 0.05; **P < 0.01; ***P < 0.001.

In this gene-editing study, we posited that silencing the TOP2α E19/I19 5′ SS in parental drug sensitive K562 cells would drive I19 retention and lead to the subsequent degradation [33,34] of mRNA transcripts transcribed from the edited TOP2α alleles, reduce TOP2α/170 expression levels, and result in drug resistance. In support of this hypothesis, qPCR experiments demonstrated that TOP2α/170 mRNA expression levels in K562/SSedit-1 and in K562/SSedit-2 (Fig 5A) cells were decreased compared to parental K562 cells. Both clonal cell lines contained only one wildtype TOP2α allele (Fig 3B) out of the three alleles known to be present in K562 cells [38,39]. TOP2α I19 retention was detectable in K562/SSedit-1 and in K562/SSedit-2 cells utilizing an edited-specific mut/E19/I19 boundary qPCR probe (Fig 5B). Importantly, TOP2α I19 retained mRNA levels in K562/SSedit-2 cells were 2-fold greater than those found in K562/SSedit-1 (Fig 5B and 5C) demonstrating that I19 retention increased with each TOP2α E19/I19 edited allele.

To further confirm that the CRISPR/Cas9/HDR strategy effectively increased TOP2α I19 retention, total cell lysates were taken from K562, K/VP.5, and K562/SSedit-2 cells for immunoassays using C-terminal- and N-terminal-specific TOP2α antibodies to evaluate TOP2α protein levels. As expected, in K562/SSedit-2 compared to K562 cell lysates, there was a decrease in TOP2α/170 protein levels (Fig 5D and 5E). Fig 5B and 5C indicated expression of E19/I19 mRNA transcripts in K562/SSedit-2 (and K562/SSedit-1) clones suggesting that gene editing of the TOP2α E19/I19 5’ SS increased I19 retention. If the retained I19 was processed and polyadenylated, it would encode the TOP2α/90 isoform. However, the lack of increased TOP2α/90 protein indicated that these mRNA transcripts were not subjected to IPA but rather were degraded.

Although the TOP2α/90 protein levels were not elevated in K562/SSedit-2 cells (Fig 5E), the ratio of TOP2α/90-to-TOP2α/170 protein was increased compared to parental K562 cells, likely due to maintained production of TOP2α/90 from the one remaining wild-type allele along with reduction in TOP2α/170 (Fig 5F). However, this ratio was considerably less than the increased TOP2α/90-to-TOP2α/170 protein ratio in K/VP.5 compared to K562 cells (Fig 5F) where IPA was demonstrated to result in increased TOP2α/90 expression [26].

RNA-seq results demonstrated I19 reads in K562/SSedit-2 cells through the full length of this intron, consistent with I19 retention without IPA (Fig 5G). In contrast, in K/VP.5 cells there were extensive E19 reads extending into I19 which we have previously demonstrated as I19 IPA and translation which resulted in elevated levels of TOP2α/90 and reduced TOP2α/170 (11,15,26). In separate qPCR experiments, there was a statistically significant increase in mRNA expression containing the I19/E20 boundary in K562/SSedit-2 compared to K562 cells (Fig 5H) consistent with intron 19 retention. There was also a statistically significant decrease in the I19/E20 expression in K/VP.5 compared to K562 cells (Fig 5H) consistent with IPA in K/VP.5 cells and decreased RNA seq exon 20 reads comparing K/VP.5 and K562 cells (Fig 5G). Both the RNA-seq and qPCR results are consistent with I19 retention and subsequent degradation in K562/SSedit-2 cells accounting for both decreased TOP2α/170 and a lack of increase in TOP2α/90 protein levels; elevated TOP2α/90 (Fig 5E) being a hallmark of K/VP.5 cells as a result of IPA (11,15,26).

Initial evaluation of degradative mechanism(s) for retained TOP2α I19 mRNA transcripts in K562/SSedit-2 cells was performed by addition of CHX, a known indirect inhibitor of NMD [36]. Cells were incubated in the presence or absence of CHX (100 μM, 3hours) followed by qPCR evaluation of TOP2α transcripts (using edited E19/I19, I19/E20 probes) as well as positive and negative NMD controls, SRSF3-variant 2 and SRSF3-variant 1, respectively [40,41]. As shown in Fig 5I, only the SRSF3-variant 2 positive control transcript was increased after CHX treatment. The TOP2α transcripts were reduced after CHX incubation. Results are consistent with degradation of K562/SSedit-2 TOP2α transcripts occurring by NMD independent mechanisms.

### Etoposide resistance in K562/SSedit-2 cells

**Fig 6:**
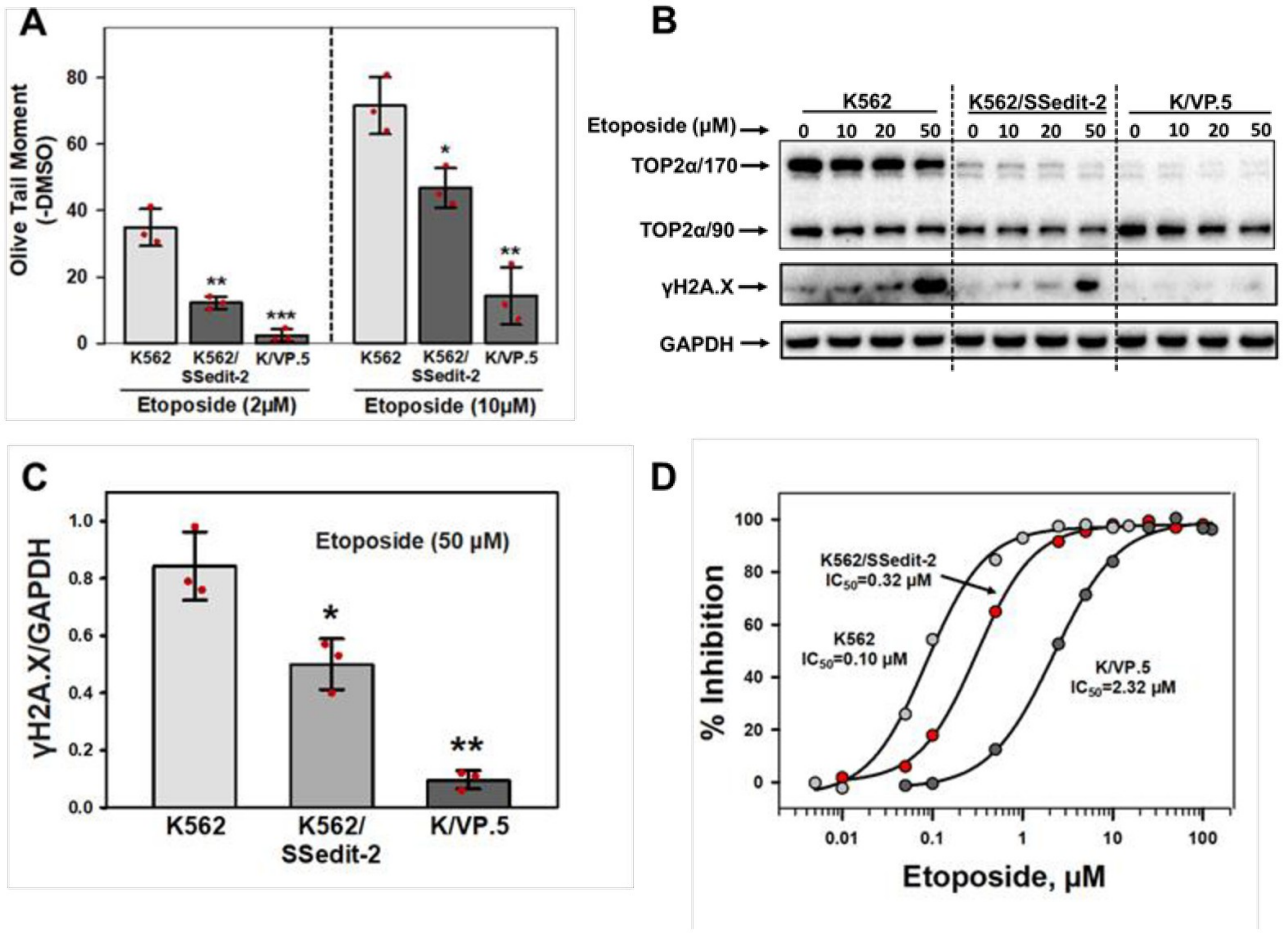
Effects of silencing the TOP2α E19/I19 5′ SS on resistance to etoposide-induced DNA damage and cytotoxicity. **(A)** K562, K/VP.5, and K562/SSedit-2 cells were incubated with etoposide (2 or 10 μM) or DMSO (control) for 30 minutes followed by alkaline (pH 13) Comet assays. Results shown are the mean ± SD for three cellular experiments run on separate days. For all experimental conditions in each experiment greater than 100 cells were evaluated by OpenComet software [35]. At 2 μM etoposide, *P* = 0.003 comparing the difference in mean values for K562/SSedit-2 vs. K562 cells, 95% CI (-32.09, -13.20); *P* < 0.001 comparing the difference in mean values for K/VP.5 vs. K562 cells, 95% CI (-41.88, -22.98). At 10 μM etoposide, *P* = 0.015 comparing the difference in mean values for K562/SSedit-2 and K562 cells, 95% CI (-41.42, -8.01); *P* = 0.001 comparing the difference in mean values for K/VP.5 vs. K562 cells, 95% CI (-76.67, -37.78). **(B)** Representative immunoblot blot from whole cell lysates of K562, K/VP.5, K562/SSedit-2 cells treated with DMSO or etoposide (10, 20, 50 μM) for 1 hour. The blot was probed with an antibody specific for the N-terminal portion of TOP2α/170/90 and with γH2AX and GAPDH antibodies. **(C)** Relative level of γH2AX at 50μM etoposide in K562, K562/SSedit-2, and K/VP.5 cells; *P* = 0.016 comparing the difference in mean values for K562/SSedit-2 vs. K562 cells, 95% CI (-0.53, -0.15); *P* = 0.007 comparing the difference in mean values for K/VP.5 vs. K562 cells, 95% CI (-1.01, -0.48). **(D)** Growth inhibitory effects of etoposide in K562, K/VP.5, and K562/SSedit-2 cells. Log-phase cells were incubated for 48 hours with various concentrations of etoposide after which cells were counted on an electronic particle counter (Z1 Dual Coulter counter). The extent of growth beyond the starting concentration in drug-treated versus DMSO controls was expressed ultimately as percent inhibition. Shown are representative concentration-response (inhibitory) curves for each of the cell lines with 50% inhibitory concentrations (IC_50_-values) indicated. Compilation of replicate experiments performed on different days is shown in Table 1. *, *P* < 0.05; **, P < 0.01, ***, *P* < 0.001.

In K562 and K/VP.5 cells, etoposide activity was directly correlated with TOP2α/170 mRNA/protein levels [11,15,26]. Since TOP2α/170 mRNA/protein levels (Fig 5A, 5D and 5E) were decreased in K562/SSedit-2 cells, it was expected that etoposide activity in these gene-edited clonal cell lines would be attenuated consistent with induction of resistance. Therefore, alkaline single cell gel electrophoresis (Comet) assays [44] were performed to assess DNA strand breaks (Olive Tail Moment) in K562, K/VP.5, and K562/SSedit-2 cells (Fig 6A). As previously demonstrated [11,15,26], etoposide-induced concentration dependent DNA strand breaks in K562 cells were decreased in resistant K/VP.5 cells (Fig 6A). In K562/SSedit-2 compared to K562 cells, there was a statistically significant decrease in the level of etoposide-induced DNA damage at both concentrations tested (2 and 10 μM) with even greater reduction in etoposide-induced DNA damage in K/VP.5 cells (Fig 6A).

Given that alkaline Comet assays detect primarily SSBs [54], etoposide-induced DNA DSBs were assessed by expression of phosphorylated H2AX (γH2AX) [55]. K562, K/VP.5, and K562/SSedit-2 cells were incubated 1 hour with DMSO solvent control or etoposide (10–50 μM) followed by lysis and immunoblotting with anti-γH2AX antibody. Etoposide induced a concentration dependent increase in expression of γH2AX in all cell lines with attenuation of DSBs in K/VP.5 cells and K562/SSedit-2 cells compared to parental K562 cells (Fig 6B and 6C). Coincident with etoposide-induced DSBs (γH2AX), there was “band depletion” of TOP2α/170 in K562, K562/SSedit-2, and K/VP.5 cells (Fig 6B). These results are consistent with etoposide induction of TOP2α/DNA covalent complexes whose large molecular weight prevents them from entering gels [56].

Next, 48-hour growth inhibition assays were performed in K562, K/VP.5, and K562/SSedit-2 cells treated continuously with etoposide. Compared to K562 cells, K562/SSedit-2 cells were 3.5-fold resistant to etoposide (Fig 6D, Table 1). In contrast, K/VP.5 cells exhibited 21.7-fold resistance (Fig 6D and Table 1) as reported previously [11,15,26]. Doubling times were similar for K562, K/VP.5, and for K562/SSedit-2 cells (Table 2) demonstrating that editing the TOP2α E19/I19 5’ SS in K562/SSedit-2 cells did not change their growth characteristics.

**Table 1:** Growth inhibitory effects of etoposide in K562, K/VP.5, and K562/SSedit-2 cells.

K562 Cells (IC_50_; nM)	K/VP.5 Cells (IC_50_; nM)	K562/SSedit-2 Cells (IC_50_; nM)	Relative Resistance^b^ (K/VP.5/ K562)	Relative Resistance^b^ (K562/SSdit-2/ K562)
**111±02 (14)** ^c^	**2,376±52**^d^ **(10)**	**384±08**^d^ **(9)**	**21.7**	**3.5**

^a^Fifty percent inhibitory concentration (IC_50_) in a 48-hour growth inhibition assay.

^b^IC_50_ of K/VP.5 or K562/SSedit-2 cells divided by that of the parental K562 cell line.

^c^Mean ± S.D.; numbers in parentheses, number of independent experiments performed on different days.

^d^Statistically significantly different compared to K562 cells.

**Table 2:** Cell line growth characteristics.

Cell Line	Doubling Time (hours)^a^
**K562**	**15.9 ± 0.5 (9)** ^b^
**K/VP.5**	**17.8 ± 0.9 (9)**
**K562/SSedit-2**	**16.2 ± 0.6 (4)**

^a^Calculated from log-linear regression plots over 3–4 days of growth.

^b^Mean ± S.D.; numbers in parentheses, number of independent experiments performed on different days.

### Overexpressing TOP2α/90 in K562/SSedit-2 induces further resistance to etoposide

**Fig 7:**
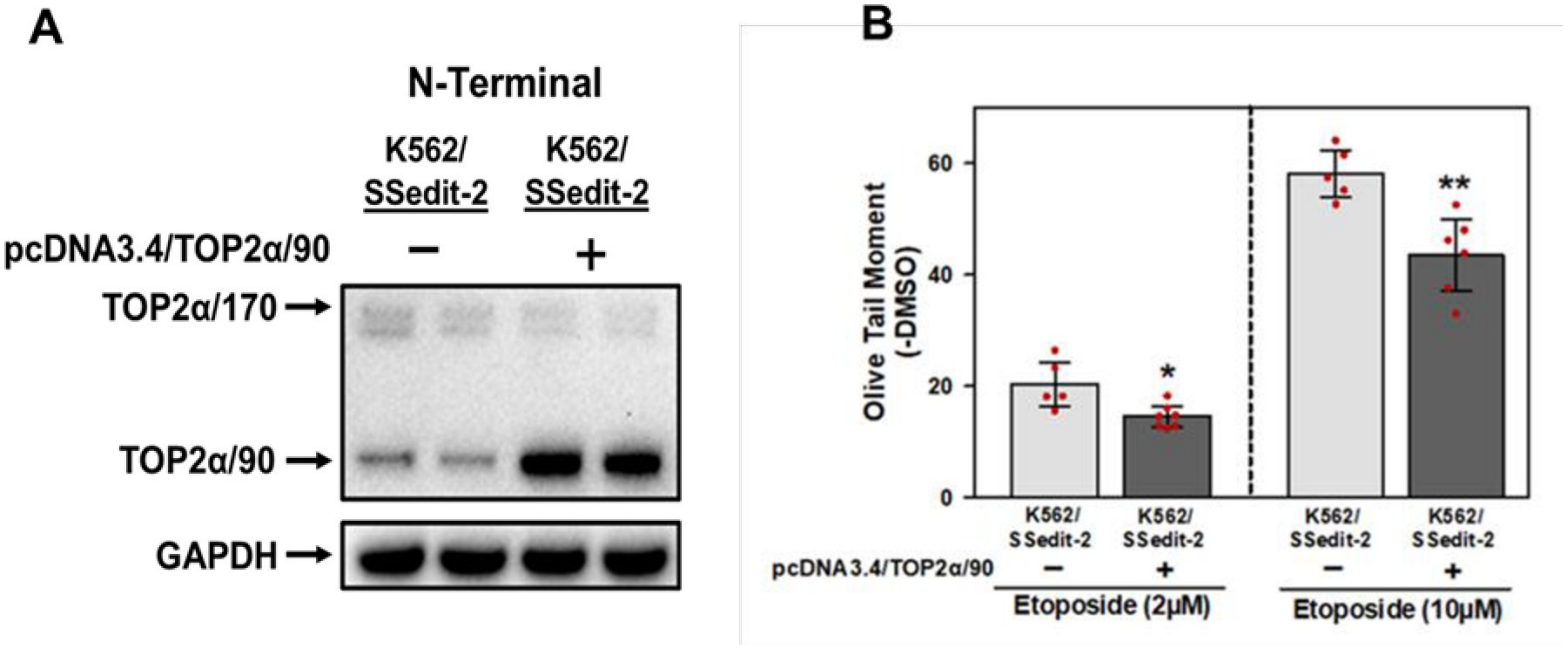
Forced expression of TOP2α/90 in K562/SSedit-2 cells further reduces etoposide sensitivity. **(A)** Representative immunoassay using cellular lysates isolated from K562/SSedit-2 mock transfected or K562/SSedit-2 pcDNA3.4/TOP2α/90 transfected cells. **(B)** K562/SSedit-2 mock transfected and K562/SSedit-2 pcDNA3.4/TOP2α/90 transfected cells were incubated with etoposide (2 or 10 μM) or DMSO (control) for 30 minutes followed by alkaline (pH 13) Comet assays. Results shown are the mean ± SD for 5 cellular experiments run on separate days. For all experimental conditions in each experiment greater than 100 cells were evaluated by OpenComet software [35]. At 2 μM etoposide, *P* = 0.012 comparing the difference in mean values for K562/SSedit-2 pcDNA3.4/TOP2α/90 transfected vs. K562/SSedit-2 mock transfected cells, 95% CI (-10.01, -1.59). At 10 μM etoposide, *P* = 0.003 comparing the difference in mean values for K562/SSedit-2 pcDNA3.4/TOP2α/90 transfected and K562/SSedit-2 mock transfected cells, 95% CI (-23.05, -6.22). *, *P* < 0.05; **, *P* < 0.01.

Although the ratio of TOP2α/90-to-TOP2α/170 protein was increased in K562/SSedit-2 cells compared to parental K562 cells (~15 fold; Fig 5F), the ratio of TOP2α/90-to-TOP2α/170 protein in etoposide resistant K/VP.5 compared to K562 cells was considerably higher (~100 fold; Fig 5F) as was resistance to etoposide (Fig 6D and Table 1). Therefore, to further evaluate the role of TOP2α/90, K562/SSedit-2 cells were transfected with a pcDNA3.4/TOP2α/90 expression plasmid. Forced-expression of TOP2α/90 in K562/SSedit-2 cells was assessed 24 hours post transfection by immunoblotting with subsequent evaluation of etoposide-induced DNA damage by Comet assay. In TOP2α/90 transfected K562/SSedit-2 cells, there was an evident increase in TOP2α/90 protein with a ratio of TOP2α/90-to-TOP2α/170 protein of ~22-fold compared to a TOP2α/90-to-TOP2α/170 ratio of ~3-fold for K562/SSedit-2 cells (Fig 7A). The increased TOP2α/90 protein expression in transfected K562/SSedit-2 cells resulted in attenuated etoposide-induced DNA damage compared to mock transfected cells (Fig 7B). Our lab previously reported that overexpressing TOP2α/90 in etoposide-sensitive K562 cells resulted in decreased etoposide-induced DNA damage attributable to heterodimerization with TOP2α/170 [11,15]. Hence, Fig 7B results further support the role of TOP2α/90 as a determinant of resistance to etoposide.

## Discussion

Our laboratory has previously established that acquired resistance to etoposide in a clonal K562 subline, K/VP.5 is associated with increased expression of a novel C-terminal truncated TOP2α isoform, TOP2α90, resulting from alternative RNA processing [11,15,16]. Specifically, this truncated isoform is translated from a mRNA that is prematurely terminated and polyadenylated ~300 nt downstream from the beginning of a retained I19 (i.e., I19 IPA) [11,15,16]. Importantly, the TOP2α/90 isoform, lacks the active site Tyr805 required to form TOP2α–DNA covalent complexes, heterodimerizes with full-length TOP2α/170, and is a determinant of resistance to etoposide and other TOP2α-targeted agents [11,15,16].

Alternative splicing is a means by which a precursor mRNA (pre-mRNA) is transcribed from a single gene and processed into multiple mature mRNAs which profoundly enhance the transcriptomic and proteomic diversity of a cell [57–62]. High-throughput sequencing has revealed that over 95% of human genes generate at least two alternative spliced mRNA isoforms [58–60]. The patterns of alternative splicing include cassette exon inclusion/exclusion, mutually exclusive splicing of adjacent exons, switching between alternative (5′ splice or 3′ splice) sites, intron retention, and other, more complex patterns of splice site selection [61,62].

Intron retention is exemplified by the inclusion of one or more introns in mature mRNA transcripts and affects ∼80% of proteins encoded by these mRNAs [62,63]. Intron-retaining mRNA transcripts can be detained in the nucleus and processed by nuclear degradation pathways [33]. Alternatively, intron-retaining mRNAs can also be exported to the cytoplasm and subsequently degraded by NMD if these transcripts contain in-frame premature termination codons (PTCs) [61–63]. In contrast, some intron-retaining mRNAs exported to the cytoplasm can undergo translation to produce new protein isoforms which are distinct from the canonical protein with novel functions and/or location [64–74].

Additionally, IPA (intron retention, RNA cleavage, and subsequent polyadenylation of the intron) can also lead to novel protein isoforms or truncated mRNA transcripts without apparent functions [75–85]. Roughly 20% of human genes have at least one IPA event [17,18] which is generally associated with weak 5′ SS, large introns, and strong polyA sequences located ~100–1000 bp downstream from the 5′ SS of an intron [17]. Regardless of the whether complete intron retention or IPA occurs in the mRNA transcript, the expression of the full-length parental protein will be reduced.

IPA is readily apparent in the case of K/VP.5 cells (Fig 5G) [26] where splice site analysis [37] of the human TOP2α gene revealed that the E19/I19 5′ SS (GAG//GTAAAC) is weak/sub-optimal with a splice score of 6.1, half the canonical maximum of 12.1 [37], contains a large I19 (1,097 bp) and harbors six strong polyA sequences [86]. Full length TOP2α/170 mRNA/protein is reduced along with enhanced expression of a novel TOP2α/90 mRNA/protein isoform [11,15,16,26]. Altogether, K/VP.5 cells display all the documented parameters for IPA [17].

Recent studies have demonstrated that the strengthening of a weak or sub-optimal 5′ SS through mutagenesis modulated alternative mRNA splicing patterns and IPA [19–25]. Previously, our laboratory utilized CRISPR/Cas9/HDR [27–32] to introduce two specific nucleotide changes (GAG//GTAAAC→GAG//GTAAGT) in the human TOP2α gene’s suboptimal E19/I19 5′ SS to improve the splicing score to 11.9 and subsequently decrease I19 IPA [26]. Remarkably, gene-edited etoposide resistant K/VP.5 cells displayed decreased TOP2α/90 mRNA/protein, with restored levels of full-length TOP2α/170 mRNA/protein and led to increased etoposide-induced DNA damage and enhanced growth inhibitory effects of this and other TOP2-targeting drugs [26]. Together, these results indicated that optimization of the TOP2α E19/I19 5′ SS in K/VP.5 cells by gene-editing decreased IPA and circumvented etoposide resistance thereby confirming the importance of IPA in acquired drug resistance to TOP2α-targeted drugs [26].

In the current study, we undertook the opposite strategy to test the hypothesis that utilizing gene-editing to silence the TOP2α E19/I19 5′ SS in parental K562 cells would interfere with normal spliceosome function resulting in TOP2α I19 retention and subsequent degradation of the edited mRNA transcripts (i.e., gene knockout) and induce drug resistance. To mutate the E19/I19 5′ SS in K562 cells and make it unrecognizable by the spliceosome, CRISPR/Cas9/HDR was utilized to make 4 changes to this boundary region to reduce the splicing score [37] from 6.1 to a -11.9 (Fig 1).

After transiently transfecting K562 cells with sgRNA-1, Cas9 along with an HDR repair template, clones were selected by limiting dilution. Successfully edited cells were initially identified by genomic qPCR and verified by sequencing (Fig 3). In addition, editing the E19/I19 5′ SS in K562 cells introduced a restriction site for the endonuclease MseI, which was utilized to further validate that isolated clones had been successfully edited by CRISRP/Cas9 (Fig 4). Two K562 clonal sublines containing one or two edited alleles of the TOP2α gene (out of three total alleles present in K562) were designated K562/SSedit-1 and K562/SSedit-2, respectively. Using custom qPCR probes across the mutated E19/I19 border of generated cDNAs indicated 2-fold greater TOP2α I19 retained mRNA expression in K562/SSedit-2 compared to K562/SSedit-1 cells (Fig 5B and 5C) consistent again with successful gene-editing of two alleles in K562/SSedit-2 cells but only one allele in K562/SSedit-1 cells. A coordinate decrease in TOP2α/170 mRNA was observed in both K562/SSedit-1 and K562/SSedit-2 cells assessed by qPCR across the E19/E20 border of generated cDNAs (Fig 5A). Compared to K562 cells, the level of TOP2α/170 mRNA in K562/SSedit-2 cells was reduced to a similar level as K562/SSedit-1 cells (Fig 5A), likely because both cell lines only have one allele capable of generating TOP2α/170. As expected, TOP2α/170 protein was also reduced in gene-edited K562/SSedit-2 cells compared to parental K562 cells (Fig 5D and 5E). Since etoposide activity is directly related to TOP2α/170 mRNA/protein [11,15,26], etoposide activity in these gene-edited clonal cell lines was expected to be attenuated consistent with induction of resistance.

Drug resistance was determined by a reduction in etoposide-induced single (Comet assay) (Fig 6A) and double (γH2AX) strand DNA breaks (Figs 6B and 6C) as well as by etoposide-induced growth inhibition in K562/SSedit-2 cells compared with parental K562 cells (Fig 6D and Table 1). These results confirmed that gene-editing to silence the E19/I19 5′ SS translated to a resistance phenotype, although the 3.5-fold etoposide resistance in K562/SSedit-2 cells was considerably less than the 21.5-fold resistance found in K/VP.5 cells (Fig 6D and Table 1). We speculated that this difference was due to the lack of increase of TOP2α/90 protein in K562/SSedit-2 cells (Fig 5E) since it was previously demonstrated that elevated TOP2α/90 in K/VP.5 cells heterodimerized with TOP2α/170 and acted as a dominant negative for etoposide activity [15].

To further confirm that TOP2α/90 protein was a direct resistance determinant, K562/SSedit-2 cells were transfected with a pcDNA3.4/TOP2α/90 expression construct, followed by assessment of etoposide-induced DNA damage. The transfection resulted in increased expression of TOP2α/90 protein (Fig 7A) and a reduction in the level of DNA damage induced by etoposide compared to mock transfected cells (Fig 7B). Importantly, our laboratory previously demonstrated that TOP2α/90 lacks the active site Tyr805 required to form TOP2α–DNA covalent complexes and heterodimerizes with TOP2α/170 [15]. Therefore, results presented here support and complement our previous results that TOP2α/90 expression decreases drug-induced TOPα-DNA covalent complexes and acts as a determinant of chemoresistance through a dominant-negative effect related to heterodimerization with TOP2α/170.

Silencing of the TOP2α E19/I19 5′ SS in parental drug sensitive K562 cells by CRISPR/Cas9/HDR attenuated TOP2α/170 (Figs 5D, 5E and 6B), and TOP2α/90 protein expression levels (Figs 5E and 6B). It is well established that mutations which interfere with exon definition by reducing 5′ or 3′ SS strength commonly result in exon skipping or intron retention [61–63,87]. Both scenarios commonly result in loss of gene function due to the translation of the miss-spliced mRNA into a nonfunctional protein or the presence of a PTC in the retained intron which would target this mRNA for degradation by NMD or a NMD independent pathway [33,34,61–63]. Interestingly, when K562/SSedit-2 cells were treated with CHX (i.e., a known indirect inhibitor of NMD [36]) TOP2α I19 retained mRNA levels were not increased (Fig 5I) suggesting that mRNA transcripts are degraded by a NMD independent pathway. We speculate that mRNA transcripts that retain TOP2α I19 may be resident in the nucleus and degraded by components of the nuclear RNA surveillance machinery [88]. Future studies will examine this possibility.

It is possible that CRISPR/Cas9 related off-target effects played a role in the induction of drug resistance observed in K562/SSedit-2 cells (Fig 6). To limit the number of off-targets, K562 cells were transiently transfected with Cas9/sgRNA-1 plus repair template, restricting the activity of these complexes [89]. Globally, CRISPR/Cas9/HDR did not affect the growth characteristics/doubling times of the edited cells compared to K562 and K/VP.5 cells (Table 2) suggesting that dramatic changes were not present. Additionally, evaluation of our RNA-seq data demonstrated that essential genes (i.e., required for cell survival) [90] were not differentially expressed (fold change cutoff of 2 at 10% false discovery rate) in gene-edited K562/SSedit-2 compared with parental K562 cells (S1 Fig, denoted in red), consistent with a lack of major off-target effects. Moreover, the top putative Cas9/sgRNA-1 off-target site/genes were predicted using the CCTop algorithm [52]. Given that more than four mismatches between the gRNA and target DNA would prevent Cas9-mediated DSB induction [26,91,92], potential sgRNA-1 off-target sites were predicted by querying the human genomic sequence (Homo sapiens GRCh38/hg38) allowing four mismatches. Importantly, none of top 20 predicted putative Cas9/sgRNA-1 off-target site/genes were differentially expressed in K562/SSedit-2 cells compared to the parental K562 cells (see GEO data). Although CRISPR/Cas9 off-target effects may have occurred in K562/SSedit-2 cells, results suggest that the predominant phenotype associated with etoposide resistance appears to be driven by the modifications in the TOP2α E19/I19 5′ SS.

In summary, CRISPR/Cas9 was utilized to make precise changes to the E19/I19 5′ SS of the TOP2α gene in K562 cells to modulate alternative mRNA splicing and induce resistance to etoposide. Forced expression of TOP2α/90 in the drug resistant K56/SSedit-2 cells further attenuated etoposide activity solidifying this isoform as a resistance determinant. Together, these data further supports the dual role of TOP2α/170 and TOP2α/90 isoforms as sensitivity/resistance determinants. Finally, these results showcase the implementation of a CRISPR/Cas9 system to further evaluate mechanisms of alternative pre-mRNA splicing and IPA and their potential role in acquired chemoresistance.

## Supporting information

S1 Fig Scatter plot of gene expression (counts per million reads mapped [CPM]) between gene-edited K562/SSedit-2 and K562 cells for 625 essential genes (denoted in blue) [83] and the top 20 putativeCas9/g-RNA-2 off-target genes predicted by the CCTop algorithm (https://cctop.cos.uniheidelberg.de/) [45] and expressed in K562 and K562/SSedit-2 cells. Dotted lines denote 2-fold change in gene expression.
(DOCX)

S1 Table Sequences of primers, single strand oligonucleotides, and qPCR hybridization probes utilized in this study. (DOCX)

S1 Raw data File containing blot/gel image data. (PPTX)
