# Supplementary figures and images for: Use of CRISPR/Cas9 with homology-directed repair to silence the human topoisomerase IIα intron-19 5’ splice site: Generation of etoposide resistance in human leukemia K562 cells
Source: PLoS One. 2022 May 26;17(5):e0265794. doi: 10.1371/journal.pone.0265794 (PMC9135202; doi:10.1371/journal.pone.0265794)

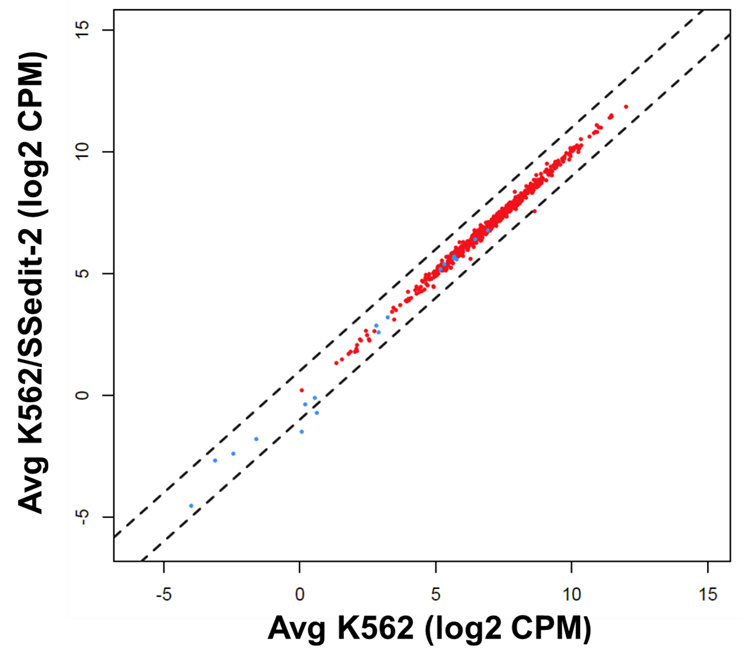

Supplement: S1 Fig — Dotted lines denote 2-fold change in gene expression. (DOCX) [file pone.0265794.s001.docx]
