# Supplementary material for: Use of CRISPR/Cas9 with homology-directed repair to silence the human topoisomerase IIα intron-19 5’ splice site: Generation of etoposide resistance in human leukemia K562 cells
Source: PLoS One. 2022 May 26;17(5):e0265794. doi: 10.1371/journal.pone.0265794 (PMC9135202; doi:10.1371/journal.pone.0265794)
Supplement: S1 Table — (DOCX) [file pone.0265794.s002.docx]

| **Primer/Oligonucleotide**  **Name** | **Primer Orientation** | **Primer/Oligonucleotide** | **Annealing Region/**  **Function** |
| --- | --- | --- | --- |
| **GCD TOP2α E18 For** | **Sense** | **5'-GATCTATCCCTTCTATGGTGG-3** | **TOP2α E18** |
| **GCD TOP2α I19 Rev** | **Antisense** | **5'-CAGAAATCAAAGGGCAAGCAG-3'** | **TOP2α I19** |
| **TOP2α guide RNA (sgRNA-1)** | **Sense** | **5'-GTCTTCTTATCATCATGGTG-3' + Scaffold** | **gRNA/Cas9 mediated DSB in TOP2α E19** |
| **Silenced E19/I19 5′ SS (4 mutations)** | **Antisense** | **5'-GAGCTTATACTTTCACCAAATCTGTTTTGAGA**  **ATGACTCTGCAGGGATTTCTGATATAATGCTTT**  **CTGGAAACATGGATTGTGTGTTAAGTTCCCCA**  **TGATGATAAGAAGACATTTCAGCCACTGATCCA**  **GCTAATTGGGCAACCTTTACTTCTCGCTTGTCA**  **TTCCGTTTGAAGCAAGT-3'** | **HDR repair template of the TOP2α E19/I19 boundary** |
| **Custom Wild type TOP2α E19/I19 boundary Taqman qPCR probe** | **Sense** | **5'-TCATGGTGAGGTAAACACACAATCC-3'** | **Wild type TOP2α E19/I19 boundary** |
| **Wild type TOP2α E19/E20 boundary Taqman qPCR probe (Assay ID Hs01032135_m1)** | **Sense** | **5'-TCATGGTGAGATGTCACTAATGATG-3'** | **Wildtype TOP2α E19/E20 boundary** |
| **Custom Silenced TOP2α E19/I19 5′ SS edited (4 mutations) qPCR Taqman probe** | **Sense** | **5'-TCATGGGGAACTTAGTACACAATCC-3'** | **CRISPR edited TOP2α E19/I19 boundary** |
