# Supplementary material for: Use of CRISPR/Cas9 with homology-directed repair to silence the human topoisomerase IIα intron-19 5’ splice site: Generation of etoposide resistance in human leukemia K562 cells
Source: PLoS One. 2022 May 26;17(5):e0265794. doi: 10.1371/journal.pone.0265794 (PMC9135202; doi:10.1371/journal.pone.0265794)
Supplement: S1 Raw data — (PPTX) [file pone.0265794.s003.pptx]

## Slide 1
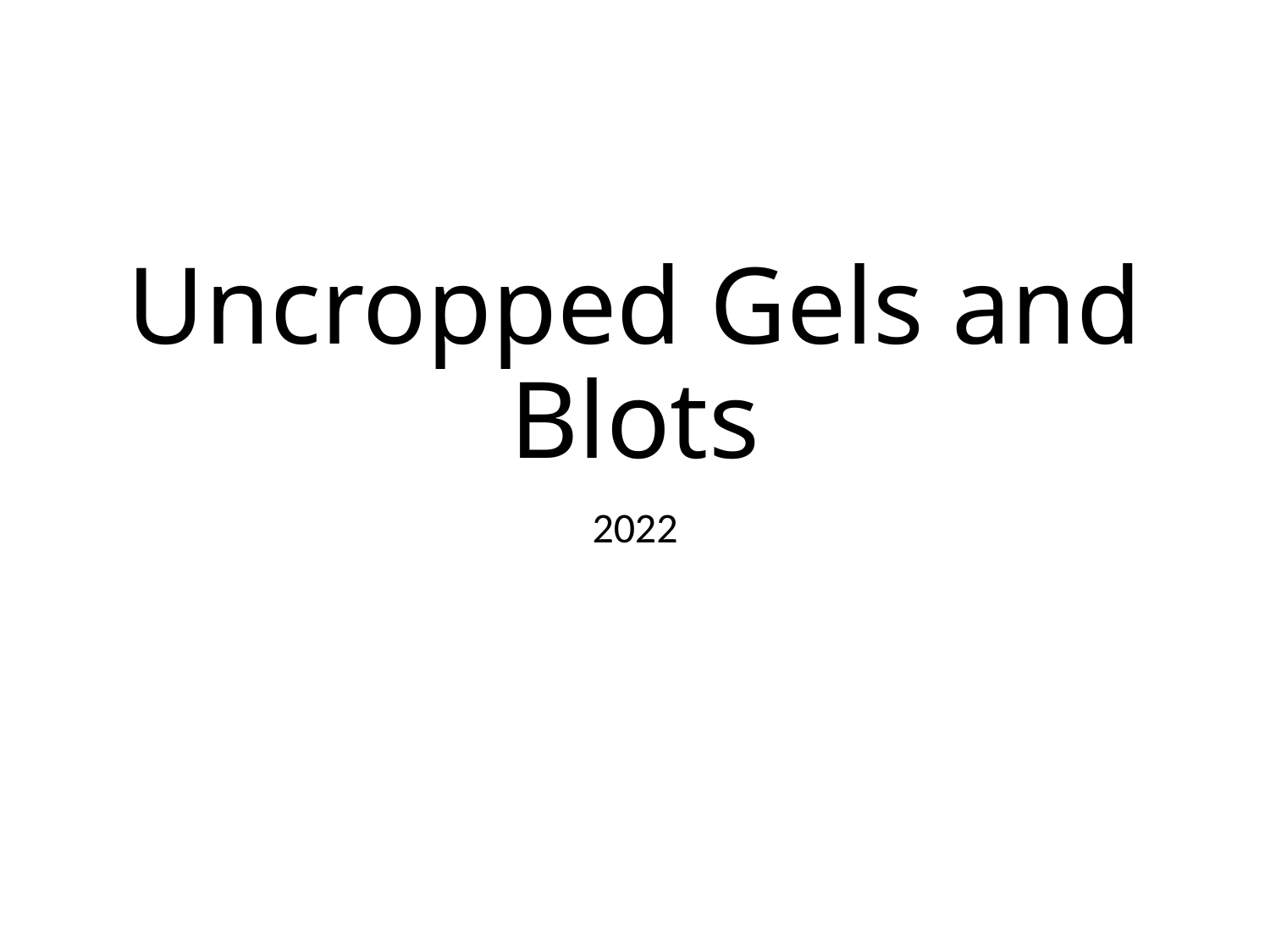

# Uncropped Gels and Blots
2022

## Slide 2
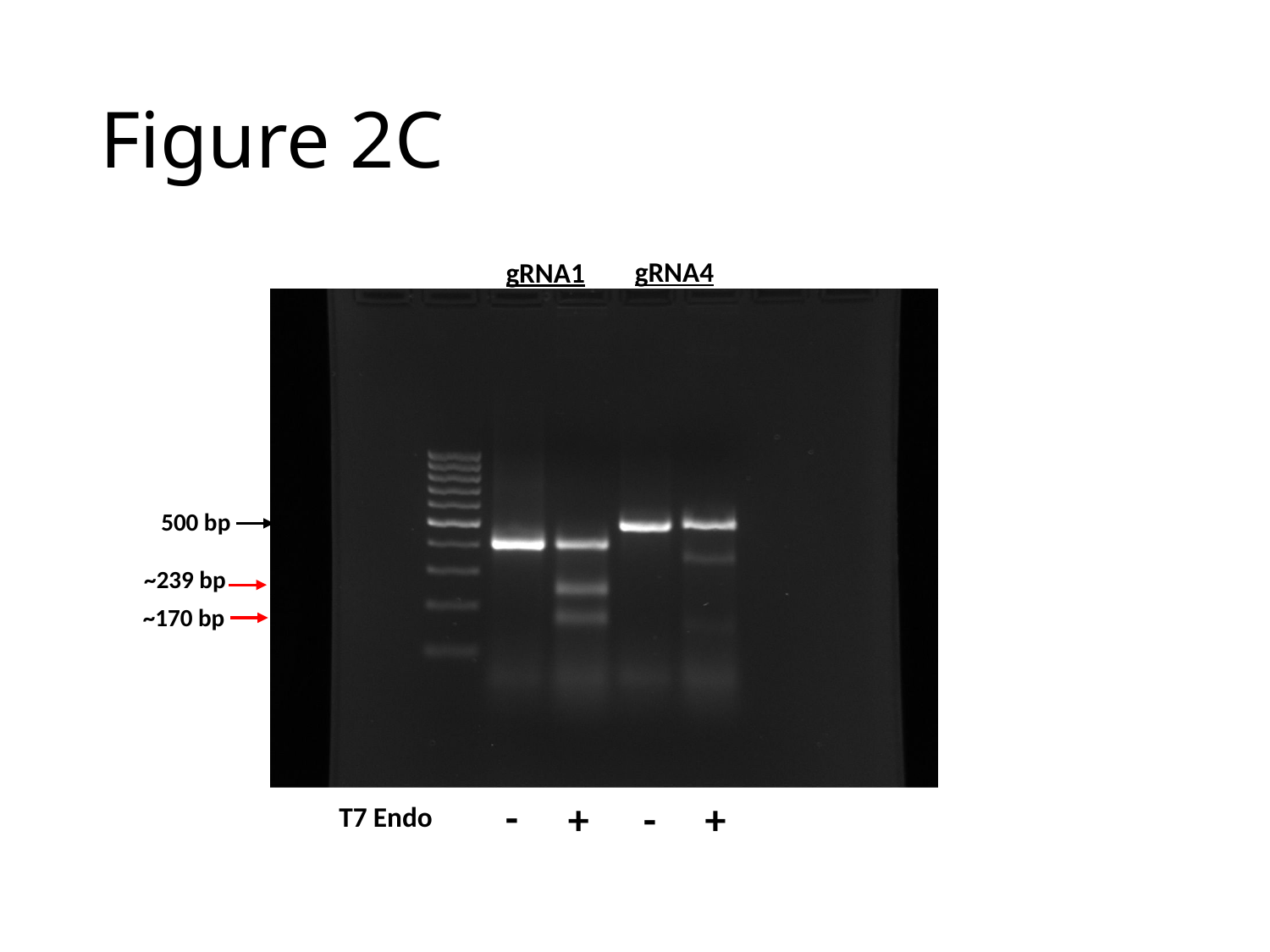

# Figure 2C
gRNA4
gRNA1
500 bp
~239 bp
~170 bp
-
+
-
+
T7 Endo

## Slide 3
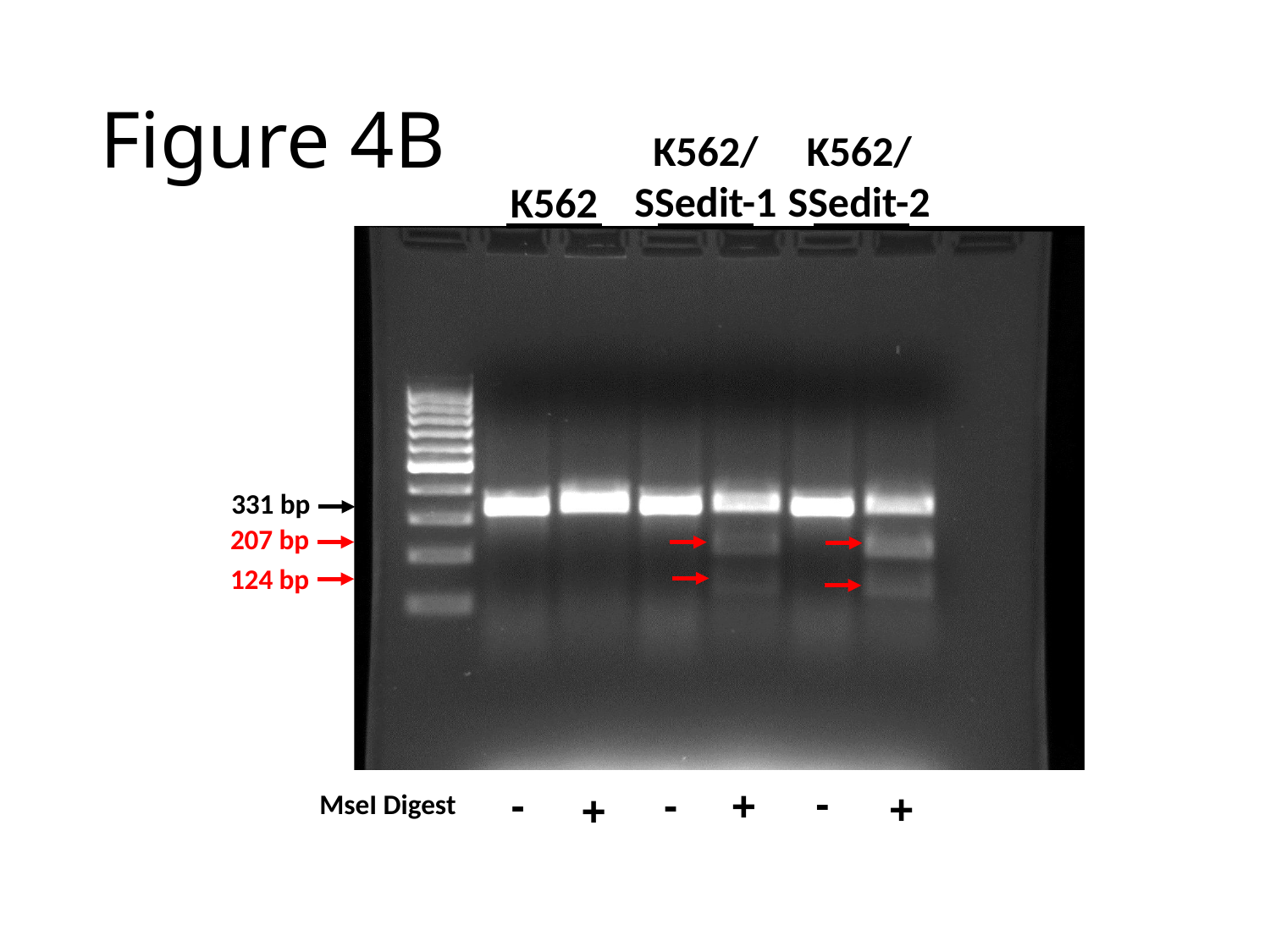

# Figure 4B
K562/
SSedit-2
K562/
SSedit-1
K562
331 bp
207 bp
124 bp
-
-
-
+
+
+
MseI Digest

## Slide 4
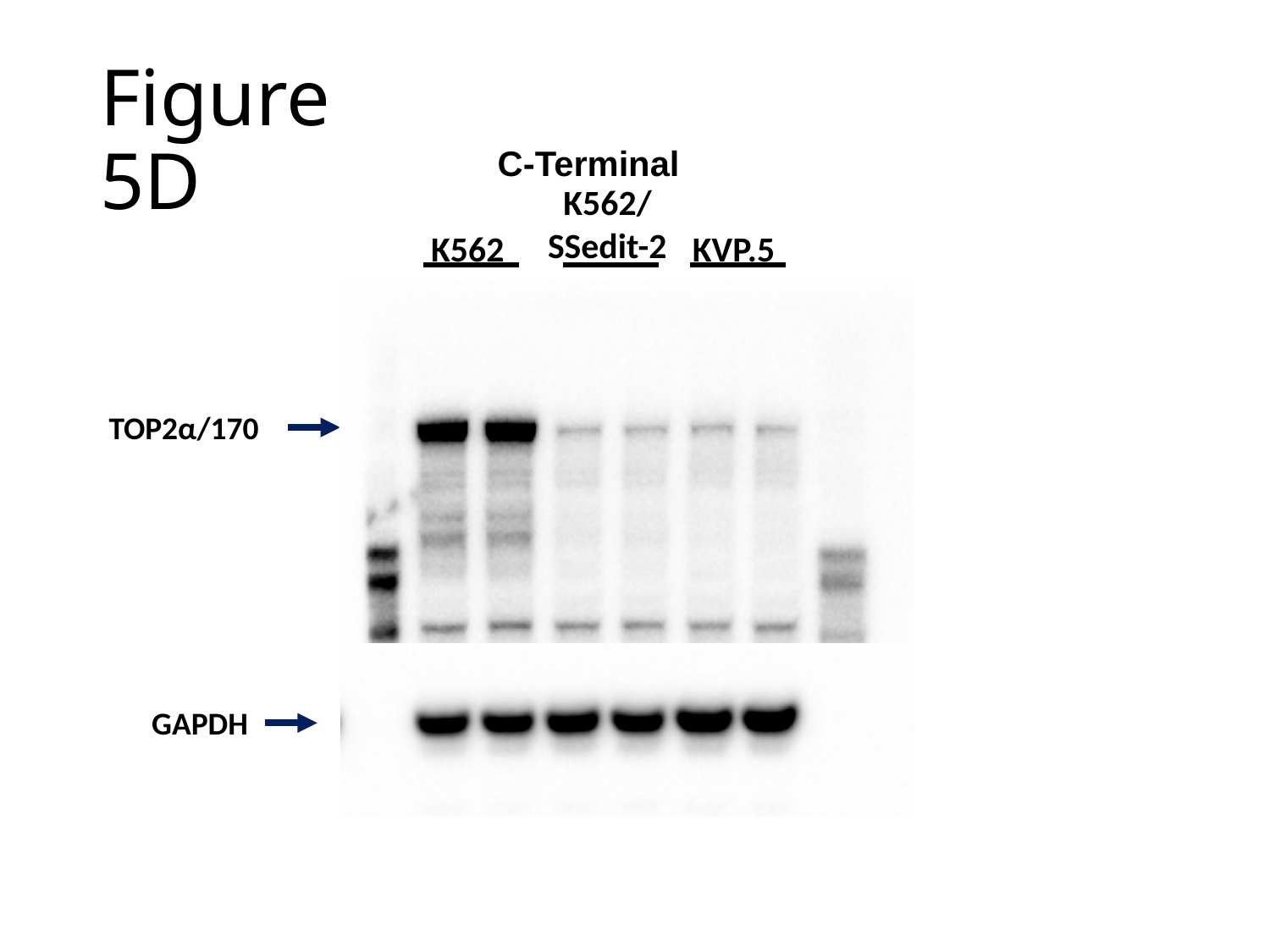

# Figure 5D
C-Terminal
K562/
SSedit-2
K562
KVP.5
TOP2α/170
GAPDH

## Slide 5
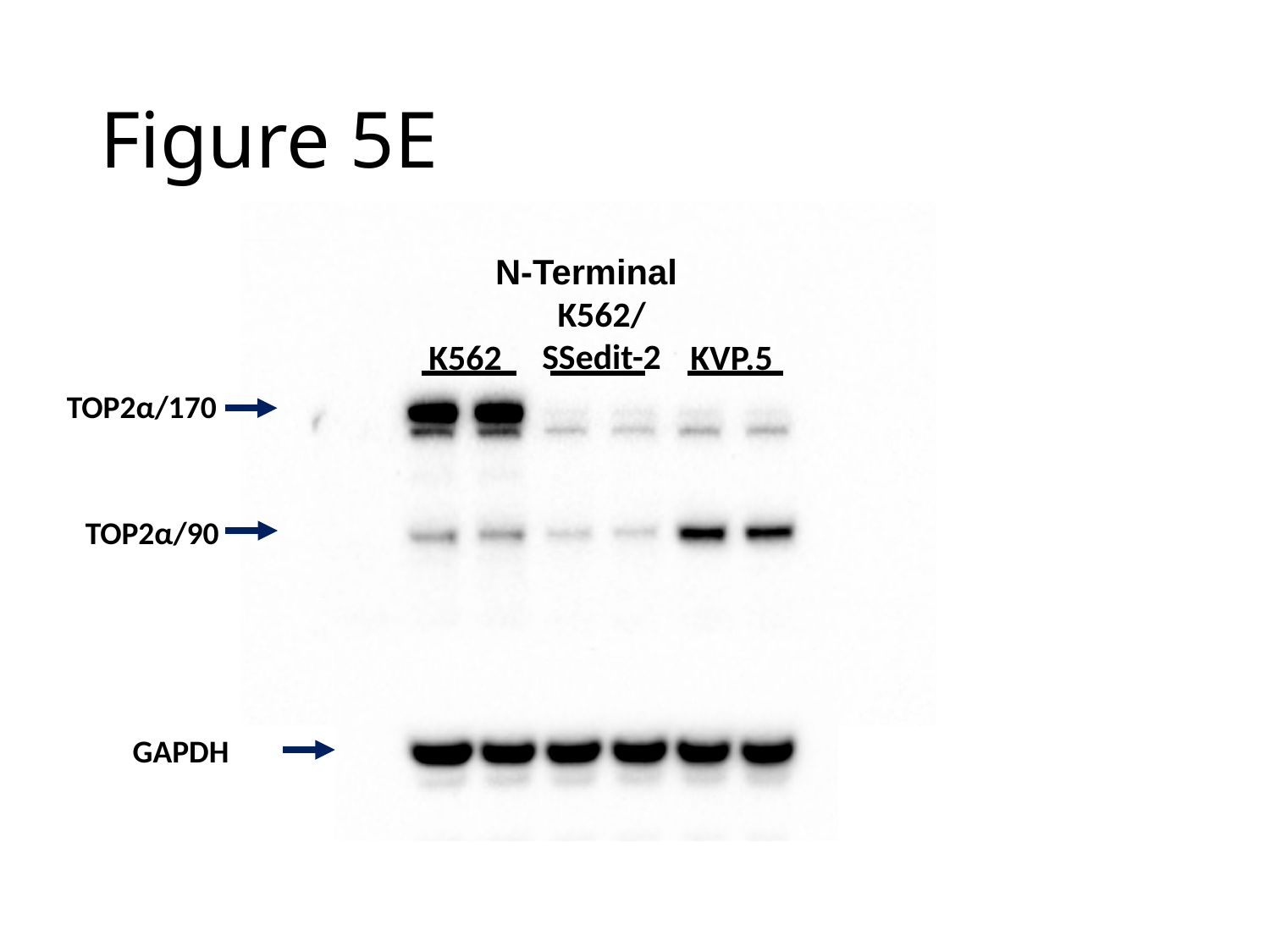

# Figure 5E
N-Terminal
K562/
SSedit-2
K562
KVP.5
TOP2α/170
TOP2α/90
GAPDH

## Slide 6
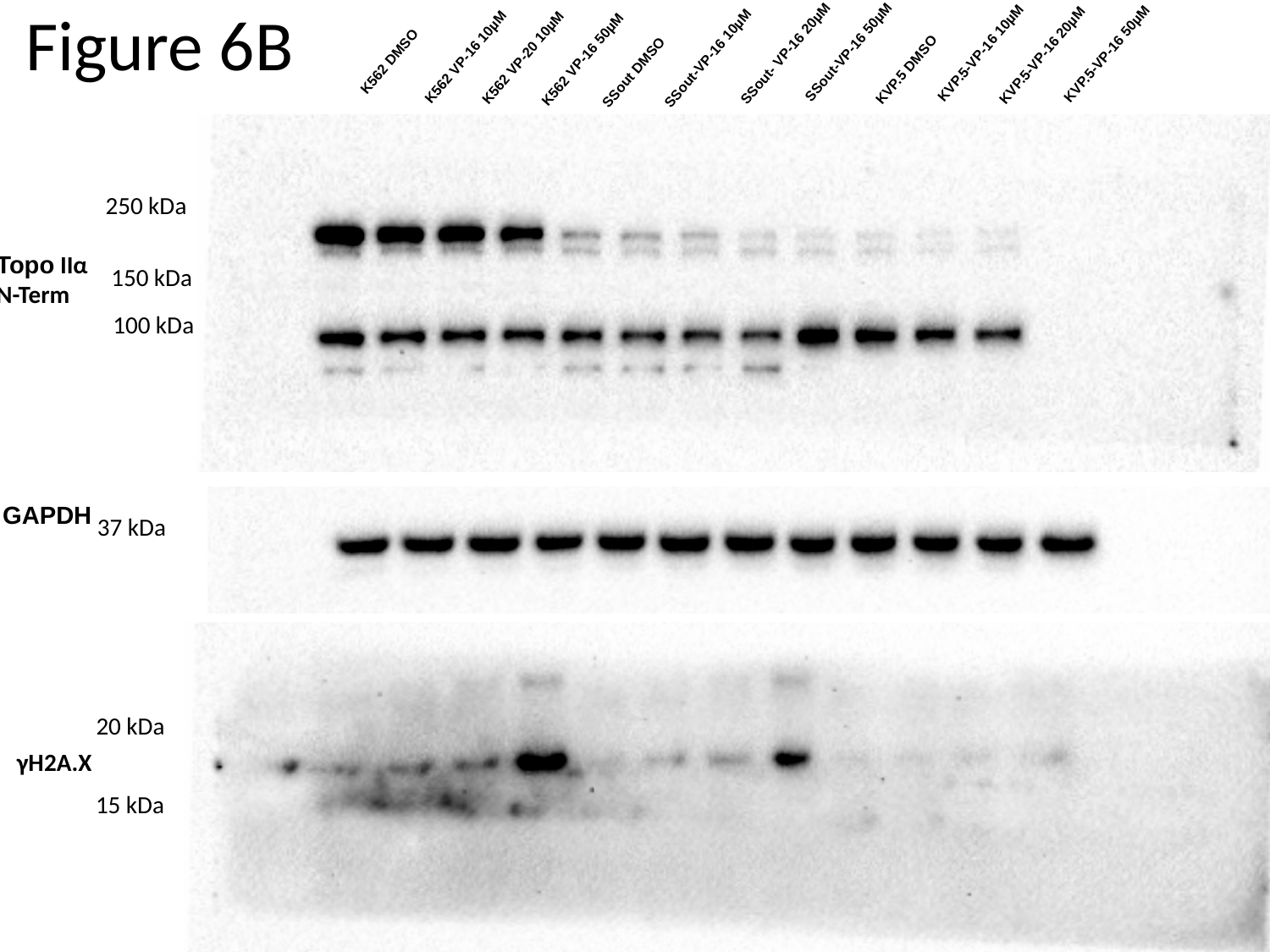

K562 DMSO
SSout-VP-16 50µM
KVP.5-VP-16 10µM
KVP.5-VP-16 50µM
KVP.5-VP-16 20µM
SSout- VP-16 20µM
K562 VP-16 10µM
SSout-VP-16 10µM
K562 VP-20 10µM
KVP.5 DMSO
Figure 6B
K562 VP-16 50µM
SSout DMSO
250 kDa
Topo IIα
N-Term
150 kDa
100 kDa
GAPDH
37 kDa
20 kDa
γH2A.X
15 kDa

## Slide 7
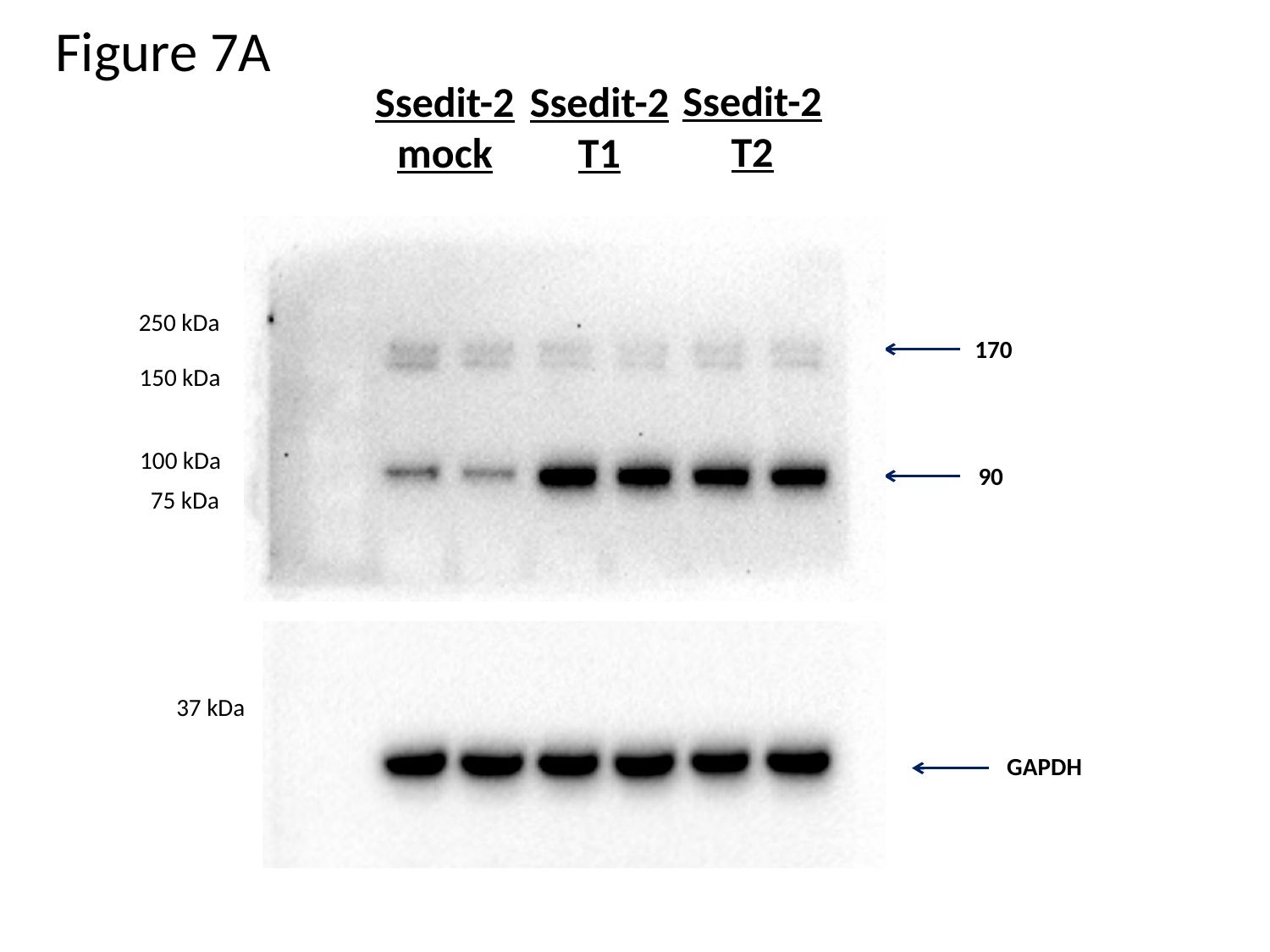

Figure 7A
Ssedit-2
T2
Ssedit-2
T1
Ssedit-2
mock
250 kDa
170
150 kDa
100 kDa
90
75 kDa
37 kDa
 GAPDH
